# Healthy Diet Metrics for Children and Adolescents and Their Suitability for Global Monitoring: A Critical Review

**DOI:** 10.1016/j.advnut.2026.100622

**Published:** 2026-03-25

**Authors:** Alissa M Pries, Hope Craig, Vrinda Mehra, Edward A Frongillo, Giles T Hanley-Cook, Chika Hayashi, Kuntal Saha, Isabela Fleury Sattamini, Teresa R Schwendler, Jennifer C Coates, Healthy Diets Monitoring Initiative

**Affiliations:** 1 Independent consultant, London, United Kingdom; 2 Friedman School of Nutrition Science and Policy, Tufts University, Boston, MA, United States; 3 Data & Analytics Section, Division of Data, Analytics, Planning and Monitoring, United Nations Children’s Fund, New York, NY, United States; 4 Department of Health Promotion, Education, and Behavior, University of South Carolina, Columbia, SC, United States; 5 Food and Nutrition Division, Food and Agriculture Organization of the United Nations, Rome, Italy; 6 Department of Nutrition and Food Safety, World Health Organisation, Geneva, Switzerland

**Keywords:** children, adolescents, diets, metrics, global monitoring

## Abstract

Healthy diets during childhood and adolescence are paramount for growth, development, and long-term health. However, there is a lack of low-burden standardized metrics to assess and monitor healthy diets among children and adolescents aged 2–19 y on a global scale. This critical review aimed to identify and evaluate existing metrics for assessing healthy diets in this age group and to determine their suitability for global monitoring based on feasibility and adaptability across different contexts. A systematic search was conducted across 3 global databases, encompassing both peer-reviewed and grey literature. A total of 127 distinct healthy diet metrics were identified, many of which were developed or adapted based on national dietary guidelines across various geographical contexts. Only 5 were deemed suitable for global monitoring due to their feasibility and adaptability: the Individual Dietary Diversity Score, 7 food group Minimum Dietary Diversity, 10 food group Minimum Dietary Diversity, Healthy Plate Variety Score, and Adapted ultraprocessed food Nova Score. Among these metrics, diversity was the most commonly measured subconstruct of a healthy diet, whereas only the Adapted Nova Score aimed to capture moderation. These 5 metrics were further evaluated for construct validity, reliability, and cross-context equivalence, which revealed large evidence gaps, particularly regarding sensitivity to change and test-retest reliability. These findings highlight the need for additional research to validate healthy diet metrics globally to ensure their accuracy, sensitivity, and reliability to differentiate populations and changes over time. Developing robust, low-burden metrics is essential for informing effective, timely nutrition policies and interventions aimed at improving the diets of children and adolescents worldwide.

**Statement of significance**

This review is the first to systematically evaluate the feasibility and adaptability of existing healthy diet metrics for global monitoring among children and adolescents, identifying key gaps in their validity and reliability, particularly regarding sensitivity to change and test-retest reliability.

## Introduction

Healthy diets during childhood and adolescence are essential for growth, development, and overall well-being [1,2]. Optimal nutrition during these periods helps to ensure children and adolescents receive adequate nutrients without excess, to prevent both undernutrition and obesity, support cognitive development, and boost immunity, to reduce the risks of infection and disease. Establishing healthy eating habits in early life sets the foundation for health in adulthood by lowering the risk of developing diet-related noncommunicable diseases, such as diabetes and heart disease, and contributing to better health outcomes throughout life [2]. Globally, countries increasingly face the triple burden of malnutrition—the coexistence of overweight and obesity, undernutrition, and micronutrient deficiencies, which stem from rapidly changing food environments, unsustainable agrifood systems, and evolving dietary choices [3,4]. Over the last 3 decades, the combined prevalence of thinness and obesity among boys and girls has increased in over 130 countries [5].

Healthy diet metrics are essential for the standardized assessment and monitoring of dietary patterns among populations across settings and over time [6]. The use of healthy diet metrics allows for the design of evidence-informed nutrition policies and interventions to improve the healthiness of diets and address nutritional gaps. Previous reviews by Marshall et al. [7] in 2014 and the subsequent update by Dalwood et al. [8] in 2020 have cataloged diet quality indices developed for use among children and adolescents. The most recent of these identified 128 unique indices and assessed their content, validity, and associations with health outcomes. These reviews provided an important foundation for understanding the landscape of diet quality metrics used among children and adolescents.

Nevertheless, significant gaps exist in our understanding of which existing measures and indicators are suitable for assessing diets among children and adolescents at scale and at regular intervals, particularly for global monitoring purposes [8]. Global monitoring of diets is critical for several reasons: it allows comparability of diets across countries and over time, supports surveillance of nutrition transitions, informs international policy frameworks, and helps track progress toward global health and sustainability goals [9]. To enable meaningful international comparisons, the use of the same or harmonized metrics across countries is essential. In addition to feasibility and adaptability, criteria such as accuracy, reliability, and demonstrated cross-context equivalence are essential for ensuring that healthy diet metrics can be validly applied across diverse cultural and socioeconomic settings. Without these features, there is a risk that monitoring data may misrepresent the healthiness of diets in certain populations. Current knowledge is limited, however, regarding the extent to which healthy diet metrics for children and adolescents perform in varied cultural and socioeconomic contexts, where dietary patterns and comprehension of questionnaires can differ substantially [10]. There is a need to: *1)* understand the spectrum of existing metrics, *2)* identify which subconstructs of a healthy diet they aim to measure, and *3)* evaluate healthy diet metrics’ applicability across diverse contexts and age groups.

In 2022, the FAO, the UNICEF, and WHO initiated the Healthy Diets Monitoring Initiative (HDMI), an effort to strengthen the knowledge base related to healthy diets metrics and their lower-burden data collection methods and to promote their uptake for high-frequency global and national monitoring. The HDMI mission statement is to “enable national and global decision-makers and stakeholders to monitor and achieve healthy diets for people and the planet” [11]. HDMI commissioned a background paper, reviewing and comparing 7 healthy diet metrics against a set of predefined assessment criteria [12]. The report, however, does not compile or assess metrics’ detailed validation findings due to time constraints and only briefly touches on the question of cross-context equivalence. Furthermore, the 7 metrics reviewed in this report were applicable to adult populations, and did not include metrics tailored to the specific nutritional requirements and dietary behaviors of children or adolescents. An important next step in evaluating the evidence base—and the innovation of this present review—is to systematically identify metrics that were designed to capture the various subconstructs of a healthy diet for children and adolescents and assess their suitability for global monitoring.

The objectives of this review were to: *1*) identify the existing metrics that have been developed or adapted to assess the subconstructs of a healthy diet among children and adolescents aged 2–19 y; *2*) assess which of these healthy diet metrics are suitable for global monitoring based on their feasibility and adaptability; and *3*) summarize the accuracy, test-retest reliability, sensitivity to change, and cross-context equivalence of healthy diet metrics suitable for global monitoring.

## Methods

### Review design

A systematic search and critical review involving a multistage method was used. In the first stage, the systematic search was conducted to identify all existing healthy diet metrics and methods used among children and adolescents. Thereafter, a critical review of the identified healthy diet metrics and methods was conducted to assess their suitability for use in global monitoring based on a set of predetermined criteria. Of this subset of healthy diet metrics, literature pertaining only to these metrics was reviewed to summarize evidence on the accuracy, reliability, sensitivity, and cross-context equivalence of metrics and methods suitable for global monitoring. Accuracy included evidence on the construct validity of metrics and reliability included studies assessing the test-retest reliability of metrics. Sensitivity to change over time included studies simultaneously comparing changes in a healthy diet metric and a reference metric of dietary intake over time. Cross-context equivalence refers to whether a metric performed equivalently across various contexts, including geography and populations of varying socioeconomic levels [13]. This review covered children and adolescents aged 2–19 y, with consideration of metrics developed or adapted across age subgroups: 2–4, 5–9, 10–14, and 15–19 y. These subgroups were selected to align with developmental stages commonly used in nutrition and health research and to ensure comparability with international frameworks (e.g., UNICEF, WHO) that often use similar age bands for child and adolescent growth, nutrition, and health reporting.

### Search strategy

The systematic search was built on a systematic review conducted by Dalwood et al. [8], which summarized diet quality indices used among children and adolescents and covered literature published up to 11 January, 2019. For this review, literature identified by Dalwood et al. [8] on metric development or adaptation was included and a search for literature published since 12 January, 2019 through 13 June, 2024 was conducted to identify additional healthy diet metrics. Databases searched include 2 used by Dalwood et al. [8] (Embase and MEDLINE) and a third database to capture peer-reviewed and grey literature (Global Health). Search terms were adapted from those used by Dalwood et al. [8] and covered 2 broad categories: *1*) the target population (children and adolescents aged 2–19 y) and *2*) healthy diet metrics (Table 1). Healthy diet metrics were defined as metrics based on the intake of nutrients, foods, or food groups, or combinations, with the aim to measure 1 or more subconstructs of a healthy diet. Subconstructs of a healthy diet included “nutrient adequacy,” “nutrient density,” “macronutrient balance,” “diversity,” “moderation,” “favorable dietary pattern,” and “food safety” [12,14]; further subconstructs of a healthy diet for children and adolescents measured in the identified metrics were also captured. Subconstructs were extracted whether stated explicitly by the study’s authors or identified implicitly by the full-text reviewers. Operational definitions of these subconstructs can be found in Supplemental Table 1.

**TABLE 1:** Systematic search terms.

Category	Search terms used
1	(child∗ or adolescent∗ or teen∗ or youth∗ or kid∗ or pre-teen∗ or minor∗).tw
2	exp child/ or exp adolescent/
3	1 OR 2
4	(“diet quality” or “dietary quality” or “dietary variety” or “diet metric” or “diet measure∗” or “dietary measure∗” or DQI or DQI-I or “Healthy Eating Index” or HEI or YHEI or “Recommended Food Score” or RFS or “variety score” or “variety ind∗” or “diversity score” or “diversity ind∗” or DDI or ACARFS or KIDMED or DGI CA or FVI or E-KINDEX or AMQI or DQQ or “diet quality questionnaire” or GDR or “global dietary recommendation∗” or “NCD-protect” or “NCD-risk” or “Nova UPF score” or GDQS or “global diet quality score” or MDD or “minimum dietary diversity”).tw
5	3 AND 4
6	Limit to 2019–present, English language only

Abbreviations: AMQI, Adolescent Micronutrient Quality Index; ACARFS, Australian Child and Adolescent Recommended Food Score; DDI, Daily Diversity Index; DGI CA, Diet Quality Index Dietary Guideline Index for Children and Adolescents; FVI, Food Variety Index; E-KINDEX, Electronic Kids Dietary Index; KIDMED, Mediterranean Diet Quality Index; UPF, ultraprocessed food.

### Inclusion and exclusion criteria

Studies were included if they report the development, adaptation, or validation of a healthy diet metric involving a population of children and adolescents aged 2–19 y (Table 2). To ensure that the review focused specifically on metrics developed, adapted or validated for the specific nutritional requirements and dietary behaviors of children and/or adolescents, studies were excluded if they involved a wider age range than 2–19 y and did not disaggregate results for children and adolescents within 2–19 y. All identified literature, both from Dalwood et al. [8] and from the updated systematic search, were subject to the inclusion and exclusion criteria.

**TABLE 2:** Inclusion and exclusion criteria.

	Inclusion criteria	Exclusion criteria
Population	Children and adolescents aged 2.0–19.9 y (sample can have a wider age range but must present disaggregated results for an age range within this window)	Samples involving only infants and young children aged 0–23.9 mo or adults aged ≥20 y
Language	English	Non–English
Scope	Report the development, adaptation or use of a healthy diet metric	Metrics where a substantial component is not related to dietary intake; metrics evaluating intake of a single nutrient, food, beverage, or food group
Reference type	Peer-reviewed publications, reports, grey literature (i.e., reports, working papers, white papers)	Abstracts; reviews that did not cover development or adaptation of healthy diet metrics

### Screening

Covidence—a systematic review management software—was used to identify duplicate references and for the screening process. After deduplication, titles and abstracts of all citations were screened for eligibility, then full texts of eligible citations were reviewed for final inclusion. Title and abstract screening and full-text review of each paper was conducted by 1 of 2 researchers (AP and HC). Double coding of 5% of papers was initially conducted to ensure consistency in screening between the 2 researchers before moving to single coding; consistency between the 2 reviewers in both abstract and full-text screening was high (98.6%). Reference lists of eligible literature were manually searched to identify any further literature for inclusion.

### Data extraction

To facilitate the multiphased method of this review, during the full-text screening process, included papers were tagged within Covidence to indicate if they contained details on: *1*) metric development, *2*) metric adaptation, *3*) predictive capacity of a metric, and/or *4*) all other forms of validity or accuracy of a metric. There were 2 phases of data extraction. Data were first extracted from all papers tagged as containing details on metric development or adaptation to identify existing healthy diet metrics for children and adolescents. These metrics were then assessed for suitability for high-frequency global monitoring (details below). In the second phase of data extraction, data were extracted from papers tagged as containing details on predictive capacity or other forms of validity/accuracy of a metric, but only among studies that reported on the predictive capacity, validity, or accuracy of the subset of metrics suitable for global monitoring. For both phases, data were extracted by 1 researcher (AP).

### Assessment of suitability for global monitoring

All healthy diet metrics for children and adolescents were assessed for suitability for global monitoring based on their feasibility and adaptability [12]. Details on the operationalization of the criteria used to assess feasibility and adaptability can be found in Table 3. The assessment of feasibility considered a metric’s ease of computation and interpretation for the subconstruct measured and ease of data collection, whereas adaptability considered the universality of the evidence underlying a metric and a metric’s application across multiple country contexts. To manage the large number of identified metrics and the limited information reported in the literature on the feasibility of data collection, the evaluation was conducted in stages. Metrics first needed to demonstrate medium or high ease of computation, high ease of interpretation, and high adaptability to be considered potentially suitable for global monitoring. This first screening reduced the pool of metrics to a manageable number for expert review and was conducted by 1 researcher (AP) based on details provided in the papers. This subset of suitable metrics was then further evaluated for the feasibility of data collection, specifically considering the burden on interviewers and respondents in terms of time, cognitive effort, and invasiveness. Cost is a key component of data collection feasibility; however, cost is highly dependent on the methods used for data collection; therefore, interviewer and respondent burden were considered as proxies for cost, where higher degrees of burden for both are typically associated with higher costs for interviewer training and data collection time. Due to the limited availability of data collection feasibility details in the literature identified through the systematic search, 4 experts in the field of child and adolescent dietary assessment were purposively and individually contacted to gather additional insights about data collection feasibility based on their experience with population-based dietary assessment methods. Consultation with these experts was conducted via email and through virtual correspondence. Each expert was provided with the same subset of shortlisted metrics and asked to comment specifically on the anticipated level of interviewer and respondent burden for each metric. Expert input was synthesized across respondents and applied using predefined criteria (Table 3). If a metric within this subset was rated as low or medium for both interviewer and respondent burden by all experts, it was considered suitable for global monitoring. This staged screening ensured that expert input focused on a small set of metrics with potential suitability, whereas feasibility of data collection remained a core, final determinant of whether a metric was considered appropriate for global monitoring. One researcher (AP) conducted the consultations for expert input and used this input to make final determinations of metric suitability for global monitoring.

**TABLE 3:** Criteria to assess suitability of healthy diet metrics for global monitoring.

Criteria	Operationalization	Scoring	Hierarchy of consideration
Feasibility—ease of computation	○ High: points assigned per respondent; score calculated; indicator tabulated ○ Medium: food and ingredients assigned to the appropriate metric item; points assigned per respondent; score calculated; indicator tabulated ○ Low: dietary data processed to estimate gram consumption of food and ingredients per respondent; food and ingredients assigned to the appropriate metric item; points assigned per respondent; score calculated; indicator tabulated ○ Very low: food composition data merged; dietary data processed to estimate gram consumption of food, ingredients, and nutrients per respondent; food, ingredient and nutrient assigned to the appropriate metric item; quantity of consumption per metric item per respondent summed; points assigned per respondent; score calculated; indicator tabulated	High—3 Medium—2 Low—1 Very low—0	First criterion considered; if metric receives <2 points (i.e., 0–1 points), it is automatically removed from further consideration.
Feasibility—ease of interpretation of subconstructs measured	○ High: metric captures only one subconstruct or allows for disaggregated submetrics for each subconstruct (e.g., a diet quality score has 2 submetrics of “unhealthy diet score” and “healthy diet score”) ○ Low: multiple subconstructs captured within the metric and disaggregated interpretation not possible (e.g., a metric including various unhealthy food groups and healthy food groups with no disaggregation)	High—1 Low—0	Second criterion considered; if metric receives 0 points, it is automatically removed from further consideration.
Adaptability	○ For metrics based on dietary recommendations/guidelines, basis is on regional or international dietary recommendations/guidance (as opposed to national dietary guideline or localized dietary pattern)	Yes—1 No—0	Third criterion considered; if metric receives a combined score of <1 point (i.e., 0 points), it is automatically removed from further consideration.
	○ For all metrics, metric developed or adapted for >1 country	Yes—1 No—0	
Feasibility—burden of data collection	Interviewer burden: level of expertise required, and perceived physical and cognitive effort ○ Low: minimal expertise in nutrition/health required; minimal physical effort required (e.g., low interview time, no equipment beyond questionnaire); low cognitive effort (e.g., capturing data on food/food groups consumed either open-recall or list-based) ○ Medium: moderate expertise in nutrition/health required (e.g., bachelors in topics); moderate physical effort required (moderate interview time, some additional equipment required); moderate cognitive effort (e.g., portion size estimation required with images) ○ High: high expertise in nutrition/health required (e.g., masters in topics); high physical effort (high interview time, substantial equipment required); high cognitive effort (e.g., portion size estimation required with food models)	Low—2 Medium—1 High—0	Fourth criterion considered; if metric receives a combined score of <2 points (i.e., 0–1 points), it is not considered suitable for global monitoring. Subjective expert opinion used for this criterion.
	Respondent burden: degree to which a respondent perceives their participation as difficult, time-consuming or emotionally stressful. ○ Low: dietary assessment for metric requires <15 min; only one assessment is required; interview is minimally invasive ○ Medium: dietary assessment for metric requires 15–30 min; only 1–2 assessments is required; interview is somewhat invasive ○ High: dietary assessment for metric requires 30+ min; multiple assessments are required; interview is somewhat invasive	Low—2 Medium—1 High—0	

### Study quality

In line with methods used by Dalwood et al. [8], a critical appraisal was conducted among studies reporting the validity, reliability, sensitivity to change over time or cross-context equivalence of a metric. This appraisal was conducted using the Academy of Nutrition and Dietetics Quality Criteria Checklist (QCC) [15]. The QCC evaluates risk of bias in participant selection, generalizability, data collection, and analysis. Based on this evaluation, a study’s quality was rated as positive, neutral, or negative. In line with the prior review by Dalwood et al. [8], studies were not excluded if their quality was determined to be negative. Assessment of study quality was conducted by 1 researcher (AP).

## Results

**FIGURE 1:**
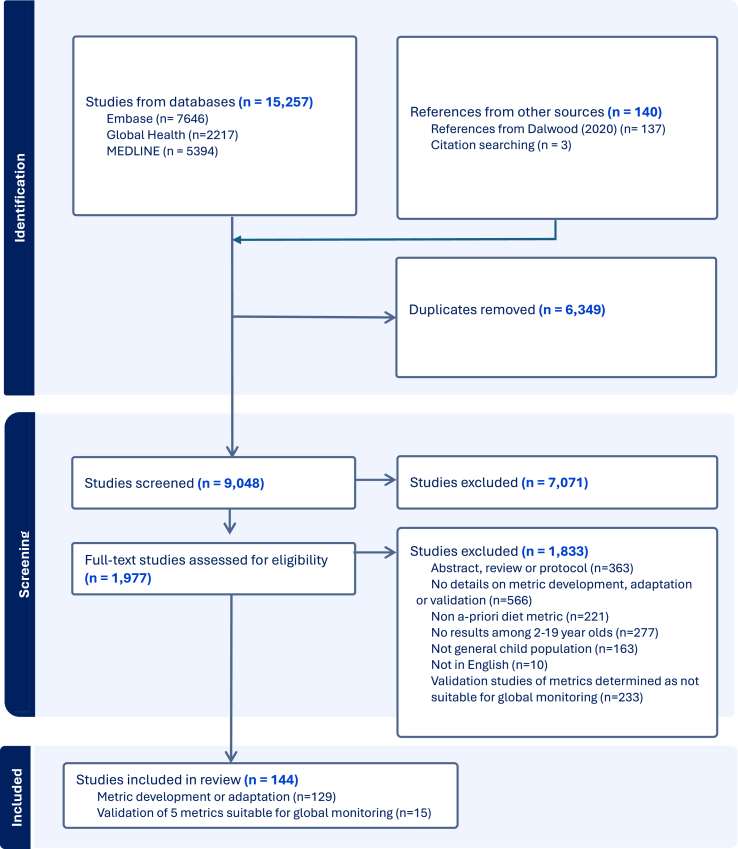
PRISMA flow diagram of study selection.

A total of 15,257 papers were identified across the 3 databases included in the systematic search (Figure 1). After deduplication and title and abstract screening, 1977 papers were included for full-text review to assess their eligibility, of which a total of 129 were included based on them containing details on the development or adaptation of a healthy diet metric for children or adolescents, from which a total of 127 metrics were identified. Fifty of these 127 metrics were additions to those identified by the review by Dalwood et al. [8], indicating that over one-third of the healthy diet metrics for children and adolescents aged 2–19 y identified by this present review have been developed or adapted for this population in the past 5 y. Of these 127 metrics, 5 were identified as suitable for global monitoring based on their feasibility and adaptability. An additional 15 papers evaluating the validity or reliability of these 5 metrics were also included in the review.

### Identified healthy diet metrics for children and adolescents

Most of the identified metrics (*n* = 103, 81.1%) were based on adherence to national dietary guidelines from a range of countries across different regions, including East Asia, Europe, Latin America, North America, Oceania, South Asia and Southeast Asia (Table 4). The 127 identified metrics were developed or adapted across 58 countries including 30 nations classified as low-, lower-middle, or upper-middle—income by the World Bank (FY24), but most of these metrics were more frequently developed or adapted for high-income countries. Metrics were commonly developed or adapted for the United States (*n =* 18), Australia (*n =* 11), Germany (*n =* 10), and Greece (*n =* 9).

**TABLE 4:** Identified developed or adapted healthy diet metrics for children and adolescents.

Metric: year of publication	Basis for metric	Subconstructs of healthy diet	Country (setting)	Ages included	Dietary assessment methods	Metric calculation and scoring	Ease of computation	Ease of interpretation	Adaptability
Revised Brazilian Healthy Eating Index (BHEI-R): 2011 [16]	Adherence to Brazilian dietary guidelines, 2004 WHO Global Strategy on Diet, 2004 IOM dietary reference intakes	Diversity (implicit) Favorable dietary pattern (implicit) Moderation (implicit) Nutrient density (implicit)	Brazil (Sao Paulo, urban)	12–19 y	Quantitative 24HR • Respondent: child • Recall: 24 h	12 components scored—9 food group-based; 3 nutrient-based; points allocated proportionately based on meeting recommendations; summed Final score: 0–100, higher score indicating healthier diet; continuous scoring only	Very low	Low	High
Diet Quality Assessment Tool for Indian Children (DQAT): 2019 [17]	Adherence to Indian dietary guidelines	Diversity (implicit) Favorable dietary pattern (implicit) Moderation (implicit) Other: meals outside the home, breakfast consumption, consumption of homemade compared with packaged foods	India (Banasthali Vidyapith, Rajasthan)	7–9 y	Quantitative 24HR • Respondent: not stated • Recall: 24 h	19 components scored—including food groups, eating behaviors and nutrient intakes; summed Final score, 0–80, higher score indicating healthier diet; continuous scoring only	Very low	Low	Low
Diet Quality Index—Costa Rica: 2020 [18]	Adherence to dietary guidelines for Costa Rica, dietary guidelines for Americans, and parameters of the Mediterranean diet and DASH diet	Diversity (implicit) Favorable dietary pattern (implicit) Moderation (implicit)	Costa Rica (location not stated)	7–18 y	Qualitative FFQ • Respondent: child • Recall: not stated	22 components scored—all food groups; points allocated based on frequency of consumption; summed Final score, 0–166, higher score indicating healthier diet; 3 categories: *1)* healthy: >119 points; *2)* requires changes: from 100 to 119 points; and *3)* unhealthy: <100 points	High	Low	High
Diet Quality Score: 2022 [19]	Adherence to United Kingdom Eatwell Guidelines	Diversity (implicit) Favorable dietary pattern (implicit) Moderation (implicit) Other: water consumption	United Kingdom (setting not stated)	7–16 y	Quantitative 24HR • Respondent: child • Recall: 24 h	11 components scored—food groups and nutrients; each scored 0–10 based on meeting recommendations; summed Final score, 0–110, higher score indicating healthier diet; continuous scoring only	Very low	Low	Low
Dietary Quality Score: 2019 [20]	On the basis of findings from systematic review of foods that increase risk of cardiovascular disease [21]	Diversity (implicit) Favorable dietary pattern (implicit) Moderation (implicit)	Argentina, Brazil, Chile, Colombia, Costa Rica, Ecuador, Peru, and Venezuela (all urban)	15–19 y	Quantitative 24HR • Respondent: child • Recall: 24 h	10 healthy components and 7 unhealthy components scored—food groups and nutrients; ordinal scores assigned based on quintiles of consumption; each summed 0–50 for healthy score and 0–35 for unhealthy score; both standardized to 100-point scale; continuous scoring only	Very low	Low	High
Chinese Healthy Eating Index for School-age Children (CHEI-SC): 2021 [22]	Adherence to 2016 dietary guidelines for China and 2013 dietary reference intakes for China	Diversity (explicit) Favorable dietary pattern (explicit) Moderation (explicit)	China (15 provinces and municipal cities)	6–17 y	Quantitative 24HR • Respondent: child (12 y and older); caregiver 6–11 y) • Recall: 24 h	19 components scored—13 healthy food groups and 6 unhealthy food groups; each component scored 0–5 or 0–10 points based on adherence to recommendations. Summed and divided by 1.6. Final score, 0–100, higher score indicating healthier diet; continuous scoring only	Very low	Low	Low
Diet Quality Index: 2021 [23]	Adherence to FAO/WHO recommendations of healthy nutrient intakes for prevention of chronic diseases	Diversity (explicit) Favorable dietary pattern (explicit) Moderation (explicit)	Mexico (Tuxtepec, Oaxaca—urban)	10–20 y	Quantitative 24HR • Respondent: not stated • Recall: 24 h	8 components scored—food groups and nutrients; each component scored 0–1 based on adherence to recommendations; summed Final score: 0–8, higher score indicating healthier diet; index terciles used to correspond to low (2–4), average (5–6) and high (7–8) preventive scores	Very low	Low	High
Diet Quality Score: 2023 [24]	Adherence to dietary guidelines for Americans	Diversity (implicit) Favorable dietary pattern (implicit) Moderation (implicit)	United States (Buffalo, New York—urban)	12–14 y	Quantitative 24HR • Respondent: child (caregiver consulted when child unsure about brand/cooking details) • Recall: 24 h	8 components scored—food groups and nutrients; scoring based on adherence to recommendations Final score, 0–8, higher score indicating healthier diet; continuous scoring only	Very low	Low	Low
Planetary Health Index: 2024 [25]	Adherence to EAT-Lancet Planetary Health recommendations	Diversity (implicit) Favorable dietary pattern (implicit) Moderation (implicit) Other: food waste	Mexico (national)	12–19 y	Semiquantitative FFQ • Respondent: not stated • Recall: 7 d	14 components scored—food groups and nutrients; each scored based on grams consumed per day from 0 to 10 points; summed Final score, 0–140, higher score indicating healthier diet; continuous scoring only	Very low	Low	High
Global Burden of Disease Index: 2024 [25]	Adherence to optimal intake estimated by the Global Burden of Disease (GBD)	Diversity (implicit) Favorable dietary pattern (implicit) Moderation (implicit)	Mexico (national)	12–19 y	Semiquantitative FFQ • Respondent: not stated • Recall: 7 d	15 components scored—food groups and nutrients; each scored based on grams consumed per day from 0 to 5 or 0 to 10 points; summed Final score, 0–120, higher score indicating healthier diet; continuous scoring only	Very low	Low	High
Mexican Dietary Guidelines Index: 2024 [25]	Adherence to 2015 Mexican dietary guidelines	Diversity (implicit) Favorable dietary pattern (implicit) Moderation (implicit) Other: water consumption	Mexico (national)	12–19 y	Semiquantitative FFQ • Respondent: not stated • Recall: 7 d	10 components scored—food groups; each scored based on portions per day from 0 to 5 or 0 to 10 points; summed Final score, 0–90, higher score indicating healthier diet; continuous scoring only	Medium	Low	Low
Diet scale: 2024 [26]	Adherence to dietary guidelines of Canary Health Service	Diversity (implicit) Favorable dietary pattern (implicit) Moderation (implicit)	Spain (Canary Islands)	6–14 y	Questionnaire • Respondent: not stated • Recall: not stated	4 components: *1)* daily intake of vegetables and ≥2 pieces of fruit per day—1 point each given, *2)* 0.5 L of dairy product intake per day—1 point given, *3)* daily intake of meat, egg or fish—1 point given, *4)* daily intake of sugar-sweetened beverages or daily intake of sweets—0 points given; summed Final score, 0–4, higher score indicating healthier diet; continuous scoring only	High	Low	Low
Food-based Diet Quality Score: 2019 [27]	Adherence to dietary recommendations for children from the Netherlands Nutrition Center and 2015 Dutch Guidelines for a Healthy Diet	Diversity (implicit) Favorable dietary pattern (implicit) Moderation (implicit)	Netherlands (Rotterdam—urban)	8 y	Semiquantitative FFQ • Respondent: not stated • Recall: 4 wk	10 components scored—food groups; ratios of reported intakes to recommended intakes (grams per day) calculated and truncated at 1; summed Final score, 0–4, higher score indicating healthier diet; continuous scoring only	Low	Low	Low
Danish Healthy Eating Index: 2019 [28]	Adherence to the Danish national food-based dietary guidelines	Diversity (implicit) Favorable dietary pattern (implicit) Moderation (explicit)	Denmark (national)	14 y	Semiquantitative FFQ • Respondent: child • Recall: 12 mo	8 components scored—food groups and nutrients; proportionally scored 0–10 based on meeting recommended intakes per day; summed Final score, 0–80, higher score indicating healthier diet; continuous scoring only	Very low	Low	Low
Diet Quality Score: 2019 [29]	Adherence to food-based dietary guidelines of the Singapore Health Promotion Board	Diversity (implicit) Favorable dietary pattern (implicit) Moderation (implicit)	Singapore (setting not stated)	6–12 y	Quantitative 24HR • Respondent: not stated • Recall: 24 h	10 components—food groups and nutrients; each scored proportionally 0–10 to reflect adherence to intake recommendations; summed Final score, 0–100, higher score indicating healthier diet; continuous scoring only	Very low	Low	Low
Swedish Healthy Eating Index for Adolescents (SHEIA15): 2020 [30]	Adherence to 2015 Swedish “Find your way” dietary guidelines	Diversity (implicit) Favorable dietary pattern (implicit) Moderation (implicit)	Sweden (national)	11–18 y	Quantitative 24HR • Respondent: child • Recall: 24 h	9 components scored—food groups and nutrients; each component calculated as ratio of actual intake to recommended intake (g) and truncated at 1; summed Final score, 0–9, higher score indicating healthier diet; continuous scoring only	Very low	Low	Low
Riksmaten Adolescent Diet Diversity Score (RADDS): 2020 [30]	Adherence to 2015 Swedish “Find your way” dietary guidelines	Diversity (implicit)	Sweden (national)	11–18 y	Quantitative 24HR • Respondent: child • Recall: 24 h	17 components scored—food groups; 1 point allocated for each food group consumed in prior day Final score, 0–17, higher score indicating healthier diet; continuous scoring only	High	High	Low
Diet Quality Score: 2020 [31]	Adherence to Greek national dietary guidelines for children and adolescents	Diversity (implicit) Favorable dietary pattern (implicit) Moderation (implicit)	Greece (national)	11, 13, and 15 y	Qualitative FFQ • Respondent: child • Recall: 7 d	4 components scored—food groups; 0–4 points allocated based on frequency of consumption in previous week; summed Final score, 0–16, higher score indicating healthier diet; continuous scoring only	High	Low	Low
Healthy Eating Score (HES): 2021 [32]	Adherence to Guatemala food-based dietary guidelines	Diversity (implicit) Favorable dietary pattern (implicit) Moderation (explicit)	Guatemala (rural)	15–19 y	Semiquantitative FFQ • Respondent: child • Recall: 3 mo	7 components scored—food groups and nutrients; proportionally scored 0–10 based on density ratio (g/2000 kcal); summed Final score, 0–70, higher score indicating healthier diet; continuous scoring only	Very low	Low	Low
Schoolchildren’s Diet Quality Scale: 2021 [33]	Adherence to Brazilian dietary guidelines	Diversity (implicit) Favorable dietary pattern (implicit) Moderation (implicit) Other: water consumption	Brazil (Florianopolis, urban)	7–12 y	Qualitative 24HR • Respondent: child • Recall: 24 h	10 components scored—food groups; each scored based on frequency of consumption with directionality for unhealthy/healthy food groups; summed Final score, 0–200, higher score indicating healthier diet; continuous scoring only	Medium	Low	Low
Malaysian Healthy Eating Index (S-MHEI): 2021 [34]	Adherence to 2013 Malaysian dietary guidelines for children and adolescents	Diversity (implicit) Favorable dietary pattern (implicit) Moderation (implicit)	Malaysia (national)	2–19 y	Information not provided	11 components scored—food groups and nutrients; each scored based on servings per 1000 kcal; summed Final score, 0–100, higher score indicating healthier diet; continuous scoring only	Very low	Low	Low
Elementary School-aged Children’s Index of Diet Quality (ES-CIDQ): 2021 [35]	Adherence to Finnish and Nordic nutrition recommendations	Diversity (implicit) Favorable dietary pattern (implicit) Moderation (implicit) Other: snacking, different eating habits on weekend days compared with weekdays	Finland (Turku, Kuopio)	8–12 y	Qualitative FFQ • Respondent: child • Recall: 7 d	15 components scored—food groups and eating behaviors; Each scored 0–2 points based on servings/behaviors per d/wk; summed Final score, 0–16.5, higher score indicating healthier diet; “poor diet” <6 points, “good diet” ≥6 points	Medium	Low	High
Chinese Preschooler Dietary Index (CPDI): 2022 [36]	Adherence to Chinese dietary guideline and Chinese dietary reference intakes	Diversity (explicit) Favorable dietary pattern (explicit) Moderation (explicit) Nutrient density (explicit)	China (12 provinces, urban and rural)	2–5 y	Quantitative 24HR • Respondent: caregiver • Recall: 24 h	11 components scored—5 healthy, 5 moderation, 1 limitation—food groups and nutrients; each score 0–2.5, 0–5 or 0–10 based on food or nutrient density per 1000 kcal; summed Final score, 0–90, higher score indicating healthier diet; continuous scoring only	Very low	Low	Low
Food-based Diet Quality Score: 2023 [37]	Adherence to 2015 Dutch guidelines for a Healthy Diet	Diversity (implicit) Favorable dietary pattern (implicit) Moderation (implicit)	Netherlands (Rotterdam—urban)	8 y	Semiquantitative FFQ • Respondent: caregiver • Recall: not stated	10 components scored—food groups; scored based on ratio of reported intakes to recommended intakes (grams per day/week) and truncated at 1; summed Final score, 0–10, higher score indicating healthier diet; continuous scoring only	Low	Low	Low
Dietary Quality Index (DQI): 2003 [38]	Adherence to dietary guidelines for an optimized mixed diet for children and adolescents	Nutrient adequacy (explicit) Moderation (implicit)	Germany (setting not stated)	2–18 y	Weighed food record • Respondent: not stated • Recall: 3 d measured	12 components scored—nutrients; scored 1 point if mean intake reaches reference intake level; reverse scoring for unhealthy nutrients; summed Final score, 0–12, higher score indicating healthier diet; continuous scoring only	Very low	Low	Low
Recommended Food Group Change Score: 1999 [39]	Adherence to dietary guidelines for an optimized mixed diet for children and adolescents	Diversity (implicit)	Germany (Dortmund—urban)	12–15 y	Weighed food record • Respondent: child • Recall: 3 d measured	Number of components differ depending on individualized recommendations; scored as ± percentage of change in servings; summed	Medium	High	Low
Total Food Group Change Score: 1999 [39]	Adherence to dietary guidelines for an optimized mixed diet for children and adolescents	Diversity (implicit)	Germany (Dortmund—urban)	12–15 y	Weighed food record • Respondent: child • Recall: 3 d measured	11 components—food groups; scored as ± percentage change in servings; summed	Medium	High	Low
Nutrient Improvement Score: 1999 [39]	Adherence to German nutrient reference values	Nutrient adequacy (explicit)	Germany (Dortmund—urban)	12–15 y	Weighed food record • Respondent: child • Recall: 3 d measured	16 components - nutrients; scored as ± percentage change in intakes	Very low	High	Low
DQI: 1988 [40]	Adherence to Canada’s Food Guide and nutrient reference values	Diversity (implicit)	Canada (Ontario)	12–16 y	Quantitative 24HR • Respondent: child • Recall: 24 h	4 components scored—food groups; scored 0–2 based on grams consumed; summed Final score, 0–8, higher score indicating healthier diet; continuous scoring only	Low	High	Low
Foods E-KINDEX: 2009 [41]	Adherence to Mediterranean dietary pattern	Diversity (implicit) Favorable dietary pattern (implicit) Moderation (implicit) Other: cooking methods	Cyprus (national)	11–13 y	Semiquantitative FFQ • Respondent: not stated • Recall: not stated	13 components scored—food groups; each scored 0–3 based on frequency of consumption in week; summed Final score, 0–37, higher score indicating healthier diet; continuous scoring only	High	Low	Low
Chinese Children Dietary Index (CCDI): 2016 [42]	Adherence to Chinese dietary guideline and Chinese dietary reference intakes	Diversity (explicit) Favorable dietary pattern (explicit) Moderation (explicit) Other: eating meals with family	China (Chengdu)	7–15 y	Quantitative 24HR • Respondent: child (9 y and older); caregiver (7–8 y) • Recall: 24 h	16 components scored—food groups, nutrients and eating behavior; each scored 0–10 based on meeting recommendation; summed Final score, 0–160, higher score indicating healthier diet; continuous scoring only	Very low	Low	Low
Healthy Dietary Adherence Score (HDAS): 2017 [43]	Adherence to common dietary guideline recommendations across all 8 countries	Diversity (implicit) Favorable dietary pattern (implicit) Moderation (implicit)	Belgium, Cyprus, Estonia, Germany, Hungary, Italy, Spain and Sweden (setting not provided)	2–9 y	Qualitative FFQ • Respondent: caregiver • Recall: 4 wk	5 components scored—food groups; each scored 0–10 based on proportion of frequency intake for each group from total intake frequency; summed Final score, 0–50, higher score indicating healthier diet; continuous scoring only	Medium	Low	High
Children’s Index of Diet Quality (CIDQ): 2015 [44]	Adherence to Nordic nutrition recommendations	Diversity (implicit) Favorable dietary pattern (implicit) Moderation (implicit)	Finland (Turku—urban)	2–6 y	Qualitative FFQ • Respondent: not stated • Recall: 7 d	13 components scored—food groups and number of daily portions; each scored based on weekly frequency of consumption; summed Final score, 0–21, higher score indicating healthier diet; continuous scoring only	Medium	Low	High
Healthy Nutrition Score for Kids and Youth: 2009 [45]	Adherence to dietary guidelines for an optimized mixed diet for children and adolescents	Diversity (implicit) Favorable dietary pattern (implicit) Moderation (implicit)	Germany (setting not stated)	3–17 y	Semiquantitative FFQ • Respondent: child (11 y and older); caregiver (3–10 y) • Recall: “last few weeks”	11 components scored—food groups; each scored based on ratio of reported intake to recommended intake based on grams per d/wk; summed Final score, 0–100, higher score indicating healthier diet; continuous scoring only	Low	Low	Low
Diet Quality Score: 2012 [46]	Adherence to dietary guidelines for an optimized mixed diet for children and adolescents	Diversity (implicit) Favorable dietary pattern (implicit) Moderation (implicit)	Germany (Munich—urban, Wesel—rural)	11 y	Semiquantitative FFQ • Respondent: caregiver • Recall: 12 mo	11 components scored—food groups; each scored based on whether intake (gram) of food amounts met recommended amounts; summed Final score, 0–11, higher score indicating healthier diet; continuous scoring only	Low	Low	Low
Healthy Lifestyle-Diet Index (HLD Index): 2010 [47]	Adherence to American Food Guide Pyramid and Canadian Food Guide	Diversity (implicit) Favorable dietary pattern (implicit) Moderation (implicit) Other: sedentary lifestyle, physical activity	Greece (setting not stated)	2–5 y	Qualitative FFQ • Respondent: not stated • Recall: not stated	11 components scored—food groups and behaviors; each scored based on frequency of consumption/behavior; summed Final score, 0–44, higher score indicating healthier diet; tertiles used to categorize 3 groups: *1)* unhealthy diet lifestyle pattern, *2)* moderate healthy diet lifestyle pattern, *3)* health diet lifestyle pattern	Medium	Low	Low
Revised Healthy Lifestyle-Diet Index (R-HLD Index): 2015 [48]	Adherence to USDA Choose My Plate	Diversity (implicit) Favorable dietary pattern (implicit) Moderation (implicit) Other: sedentary lifestyle, physical activity	Greece (Attica, Aitoloakarnania, Thessaloniki and Heraklion)	9–13 y	Quantitative 24HR • Respondent: child • Recall: 24 h (repeated 3 times to estimate weekly intake)	12 components scored—food groups and behaviors; each scored based on frequency of consumption/behavior; summed Final score, 0–48, higher score indicating healthier diet; tertiles used to categorize 3 groups: *1)* unhealthy diet lifestyle pattern, *2)* moderate healthy diet lifestyle pattern, *3)* health diet lifestyle pattern	Medium	Low	Low
Food Index (FI): 2015 [49]	Adherence to United States dietary guidelines and Mediterranean Food Pyramid guidelines	Diversity (implicit) Favorable dietary pattern (implicit) Moderation (implicit)	Greece (setting not stated)	9–13 y	Semiquantitative FFQ • Respondent: child • Recall: 12 mo	14 components scored—food groups; each scored based on whether intake (g) of food amounts met recommended amounts; summed Final score, 16–64, higher score indicating healthier diet; continuous scoring only	Low	Low	Low
Unhealthy Food Choices Score (UFCS): 2004 [50]	Adherence to United States and Greek dietary guidelines	Diversity (implicit) Favorable dietary pattern (implicit) Moderation (implicit)	Greece (urban and rural)	11.5, 13.5, 15.5 y	Qualitative FFQ • Respondent: not stated • Recall: not stated	10 components scored—food groups; each scored 1–5 based on frequency of consumption—reverse scoring for unhealthy foods; summed Final score, 9–45, higher score indicating healthier diet; continuous scoring only	High	Low	Low
Healthy Lifestyle-Diet Index (HLD Index): 2010 [51]	Adherence to USDA My Pyramid and Mediterranean dietary pattern	Diversity (implicit) Favorable dietary pattern (implicit) Moderation (implicit) Other: sedentary lifestyle, physical activity	Greece (Athens—urban)	10–12 y	Quantitative 24HR • Respondent: not stated • Recall: 24 h (repeated 3 times to estimate weekly intake)	10 components scored—food groups and behaviors; each scored 0–4 based on frequency of frequency of servings/behavior per d/wk; summed Final score, 0–40, higher score indicating healthier diet; continuous scoring only	Medium	Low	Low
Adolescent Micronutrient Quality Index (AMQI): 2010 [52]	Adherence to 2005 Indian dietary guidelines and 2005 dietary guidelines for Americans	Diversity (implicit) Favorable dietary pattern (implicit) Moderation (implicit)	India (Pune—urban)	10–16 y	Quantitative 24HR • Respondent: child • Recall: 24 h	13 components scored—food groups and variety; each scored based on daily serving recommendations (gram); summed Final score, 0–100, higher score indicating healthier diet; continuous scoring only	Low	Low	Low
Unweighted Diet Quality Score (DQS): 2015 [53]	Adherence to Irish dietary guidelines and Food Safety Authority of Ireland recommendations	Diversity (implicit) Favorable dietary pattern (implicit) Moderation (implicit) Other: water consumption	Ireland (national)	9 y	Qualitative 24HR • Respondent: caregiver • Recall: 24 h	Food group components scored based on frequency of consumption in last day; healthy receiving points and unhealthy subtracting points; summed Final score, –12–28, higher score indicating healthier diet; continuous scoring only	High	Low	Low
Korean Dietary Action Guides for Children Adherence Index (KDAGCAI): 2013 [54]	Adherence to 2009 Korean Dietary Action Guides for Children	Diversity (implicit) Favorable dietary pattern (implicit) Moderation (implicit) Food safety (implicit) Other: table manners, reading food labels, eating meals with family, regular mealtimes; food hygiene	South Korea (Seoul—urban)	3–12 y	Questionnaire • Respondent: caregiver • Recall: not stated	19 components—food group consumption and behaviors; each component scored 1–5 based on Likert scale of adherence to recommendations; 19 components averaged Final score, 0–5, higher score indicating healthier diet; continuous scoring only	High	Low	Low
Healthy Eating Index for Malaysians: 2015 [55]	Adherence to Malaysian dietary guidelines for children and adolescents	Diversity (implicit) Favorable dietary pattern (implicit) Moderation (implicit)	Malaysia (Kuala Lumpur—urban)	13–16 y	Quantitative 24HR • Respondent: child • Recall: 24 h	9 components—food groups and nutrients; each component scored 0–10 based on adherence to recommendation quantities; summed and standardized to 100 Final score, 0–100, higher score indicating healthier diet; continuous scoring only	Very low	Low	Low
DQI for NZ Adolescents (NZDQI-A): 2013 [56]	Adherence to New Zealand Food and Nutrition Guidelines for Healthy Adolescents	Diversity (implicit)	New Zealand (Dunedin—urban)	14–18 y	Qualitative FFQ • Respondent: child • Recall: not stated	5 components—food groups; each component scored 0–20 based on servings and frequency per day; summed Final score, 0–100, higher score indicating healthier diet; continuous scoring only	High	High	Low
Norwegian Adolescent Diet Score: 2016 [57]	Adherence to Norwegian dietary recommendations	Diversity (implicit) Favorable dietary pattern (implicit) Moderation (implicit) Other: water consumption, physical activity	Norway (Hordaland county)	14–15 y	Qualitative FFQ • Respondent: child • Recall: 3 mo	5 components—food groups and behavior; each component receiving 1 point if servings/practices per d/wk met recommendations; summed Final score, 0–8, higher score indicating healthier diet; continuous scoring only	High	Low	Low
Individual Dietary Diversity Score (IDDS): 2007 [58]	Adherence to diversity guidelines for developing countries	Diversity (explicit)	Philippines (national)	2–6 y	Qualitative 24HR • Respondent: child • Recall: 24 h	9 components—food groups; each component receiving 1 point if consumed in previous day; summed Final score, 0–9, higher score indicating healthier diet; continuous scoring only	Medium	High	High
Healthy Eating Index: 2014, 2019, 2023 [[59], [60], [61]]	Adherence to 2006 WHO Europe Food and Nutrition Policy for Schools	Diversity (implicit) Favorable dietary pattern (implicit) Moderation (implicit)	Portugal (Porto—urban)	4–10 y	Qualitative FFQ/semiquantitative FFQ • Respondent: caregiver • Recall: 6 mo	7–8 components—food groups; each component scored 1–4 based on quartiles [59,60] of consumption frequency or grams per day [61] (reverse directionality for unhealthy groups); summed Final score, 7–28/0–32, higher score indicating healthier diet; continuous scoring only	Medium	Low	High
Adapted Healthy Eating Index: 2020 [62]	Adherence to 2006 WHO Europe Food and Nutrition Policy for Schools	Diversity (implicit) Favorable dietary pattern (implicit) Moderation (implicit)	Portugal (national)	3–17 y	Quantitative 24HR and semiquantitative FFQ • Respondent: child (10 y and older) • Recall: 24 h Food diaries • Respondent: caregiver (3–9 y) • Recall: 24 h	9 components—food groups; each component scored 1–4 based on quartiles of consumption in grams (reverse directionality for unhealthy groups); summed Final score, 0–36, higher score indicating healthier diet; continuous scoring only	Low	Low	High
Diet Quality Score: 2009 [63]	Adherence to the Caroline Walker Trust National Dietary Recommendations for Scotland	Diversity (implicit) Favorable dietary pattern (implicit) Moderation (implicit)	Scotland (setting not stated)	2 y	Questionnaire • Respondent: caregiver • Recall: not stated	Dichotomous scoring of whether child meets 5 recommendations for portions of food groups	High	Low	Low
Mediterranean DQI for Children and Adolescents (KIDMED index score): 2004, 206, 2019 [[64], [65], [66]]/Brazilian Mediterranean DQI for Children and Adolescents (B-KIDMED index score): 2020 [67]	Adherence to Mediterranean Diet	Diversity (implicit) Favorable dietary pattern (implicit) Moderation (implicit) Other: breakfast consumption	Spain (setting not stated); United States (national); Brazil (Antonio Prado, Rio Grande do Sul)	2–19 y	Questionnaire • Respondent: not stated • Recall: not stated	16/17 components—food groups and behaviors; 12 positive components receive 1 point if met, 4 negative components subtract 1 point if met; summed Final score, –4/–5 to 12, higher score indicating healthier diet; continuous scoring only	High	Low	High
Mediterranean Diet Pattern (MDP): 2010 [68]	Adherence to Mediterranean Diet	Diversity (implicit) Favorable dietary pattern (implicit) Moderation (implicit)	Spain (Granada)	8–15 y	Semiquantitative FFQ • Respondent: not stated • Recall: not stated	8 components—food groups and nutrients; scored based on high consumption (using median intake to denote high); summed Final score, 0–100, higher score indicating healthier diet; continuous scoring only	Very low	Low	Low
Traditional Costa Rican Adolescents Diet Score: 2021 [69]	Adherence to Mediterranean Diet	Diversity (implicit) Favorable dietary pattern (implicit) Moderation (implicit)	Costa Rica (San Jose province—rural and urban)	13–18 y	Weighed food record • Respondent: child • Recall: 3 d measured	14 components—food groups; each received 1 point if met g/d intake recommendation, inverse scoring for unhealthy foods; summed Final score, 0–14, higher score indicating healthier diet; continuous scoring only	Very low	Low	Low
Mediterranean Diet Quality Index International (Med DQI-I): 2007 [70]	Adherence to Spanish recommended daily intakes	Diversity (explicit) Favorable dietary pattern (explicit) Macronutrient balance (explicit) Moderation (explicit) Nutrient adequacy (explicit)	Spain (Granada)	6–18 y	Semiquantitative FFQ • Respondent: not stated • Recall: not stated	4 major components with subcomponents: *1*) variety—variety across and within food groups; *2*) adequacy—food groups and nutrients; *3*) moderation—food groups and nutrients; *4*) overall balance—macronutrient ratios; components scored based on adherence to recommendations (based on servings, nutrient intakes, and frequency); summed Final score, 0–100, higher score indicating healthier diet; continuous scoring only	Very low	Low	Low
Youth Healthy Eating Index—Taiwan Revised (YHEI-TwR-90): 2018 [71]	Adherence to dietary guidelines for Americans	Diversity (implicit) Favorable dietary pattern (implicit) Moderation (implicit) Other: breakfast consumption, dinner consumption	Taiwan (national)	16–18 y	Quantitative 24HR • Respondent: child • Recall: 24 h	10 components scored—food groups and eating behaviors; each component scored 0–5 or 0–10 points based on daily consumption (servings) or practices; summed Final score, 0–100, higher score indicating healthier diet; continuous scoring only	Medium	Low	Low
Canadian Health Eating Index (HEI-C): 2017 [72]	Adherence to 2007 Canada’s Food Guide	Diversity (implicit) Favorable dietary pattern (implicit) Moderation (implicit)	Canada (national)	2–19 y	Quantitative 24HR • Respondent: not stated • Recall: 24 h	11 components—food groups and nutrients; each component scored proportionally based on adherence to recommendation for servings, % of TEI, and intakes; summed Final score, 0–100, higher score indicating healthier diet; continuous scoring only	Very low	Low	Low
Healthy Eating Index: 1995 [73]	Adherence to dietary guidelines for Americans and USDA Food Guide Pyramid	Diversity (implicit) Favorable dietary pattern (implicit) Moderation (implicit)	United States (national)	2–19 y	Quantitative 24HR • Respondent: not stated • Recall: 24 h	10 components scored—food groups and nutrients; each component scored proportionally based on meeting recommendations in intakes or % of TEI; summed Final score, 0–100, higher score indicating healthier diet; continuous scoring only	Very low	Low	High
Canadian Health Eating Index (HEI-C): 2006 [74]	Adherence to 1993 Canada’s Good Guide to Healthy Eating and 1990 Canadian Nutrient Recommendations	Diversity (implicit) Favorable dietary pattern (implicit) Moderation (implicit)	Canada (Nova Scotia, New Brunswick, Prince Edward Island, Newfoundland)	2–14 y	Quantitative 24HR • Respondent: caregiver • Recall: 24 h	9 components scored—food groups and nutrients; each component scored proportionally based on meeting recommendations in servings, intakes or % of TEI; summed Final score, 0–100, higher score indicating healthier diet; continuous scoring only	Very low	Low	Low
Healthy Eating Index for Brazilians: 2014 [75]	Adherence to Brazilian dietary guidelines	Diversity (implicit) Favorable dietary pattern (implicit) Moderation (implicit)	Brazil (Sao Leopoldo—urban)	3–4 and 7–8 y	Quantitative 24HR • Respondent: child (7–8 y with assistance of caregiver), caregiver (3–4 y) • Recall: 24 h	10 components scored—food groups and nutrients; each component scored proportionally based on meeting recommendations in intakes or % of TEI; summed Final score, 0–100, higher score indicating healthier diet; continuous scoring only	Very low	Low	Low
Youth Healthy Eating Index (Y-HEI): 2004 [76]	Adherence to dietary guidelines for Americans	Diversity (implicit) Favorable dietary pattern (implicit) Moderation (implicit) Other: breakfast consumption, eating meals with family	United States (national)	9–14 y	Qualitative FFQ • Respondent: child • Recall: 12 mo	13 components scored—food groups and eating behavior; each component scored 0–10 based on servings/frequency; summed and standardized to 100 Final score, 0–100, higher score indicating healthier diet; continuous scoring only	Medium	Low	High
Youth Healthy Eating Index—Taiwan: 2011, 2012 [77,78]	Adherence to dietary guidelines for Americans	Diversity (implicit) Favorable dietary pattern (implicit) Moderation (implicit) Other: breakfast consumption, eating meals with family, multivitamin use	Taiwan (national)	6–13 y	Quantitative 24HR and FFQ • Respondent: not stated • Recall: not stated	11 components scored—food groups and eating behaviors; each component scored 0–5 or 0–10 points based on daily consumption (servings) or practices; summed Final score, 0–90, higher score indicating healthier diet; continuous scoring only	Medium	Low	Low
Adapted Youth Healthy Eating Index (aYHEI): 2012 [79]	Adherence to dietary guidelines for Americans	Diversity (implicit) Favorable dietary pattern (implicit) Moderation (implicit)	Canada (Manitoba)	11–14 y	Qualitative FFQ • Respondent: child (with assistance of caregiver for questions on specific oils and fortified products) • Recall: 12 mo	10 components scored—food groups; each component scored 0–5 or 0–10 based on frequency of consumption per day/month; summed Final score, 0–85, higher score indicating healthier diet; continuous scoring only	Medium	Low	Low
Modified Healthy Eating Index (mHEI): 2018 [80]	Adherence to USDA food pyramid and dietary guidelines	Diversity (implicit) Favorable dietary pattern (implicit) Moderation (implicit)	Iran (Tehran – urban)	6–18 y	Semiquantitative FFQ • Respondent: not stated • Recall: 12 mo	10 components scored—food groups and eating behavior; each component scored 0–10 based on servings or grams per day; summed and standardized to 100 Final score, 0–100, higher score indicating healthier diet; continuous scoring only	Low	Low	Low
Adapted Youth Healthy Eating Index (A-YHEI): 2022 [81]	Adherence to United States dietary guidelines	Diversity (implicit) Favorable dietary pattern (implicit) Moderation (implicit)	United States (Massachusetts)	3 y	Qualitative FFQ • Respondent: caregiver • Recall: 4 wk	9 components scored—food groups; each component scored 0–5 or 0–10 based on servings per day; summed Final score, 0–80, higher score indicating healthier diet; continuous scoring only	Medium	Low	Low
Healthy Eating Index—2005: 2008 [82]	Adherence to dietary 2005 guidelines for Americans and MyPyramid Food Guidance System	Diversity (implicit) Favorable dietary pattern (implicit) Moderation (implicit)	United States (national)	2–19 y	Quantitative 24HR • Respondent: not stated • Recall: 24 h	12 components scored—food groups and nutrients; each component scored proportionally based on meeting recommendations in intakes or % of TEI; summed Final score, 0–100, higher score indicating healthier diet; continuous scoring only	Very low	Low	Low
Canadian Healthy Eating Index—2009 (HEIC-2009): 2010 [83]	Adherence to 2007 Eating Well with Canada’s Food Guide	Diversity (implicit) Favorable dietary pattern (implicit) Moderation (implicit)	Canada (Waterloo, Ontario—urban)	11–12 y	Quantitative 24HR • Respondent: child • Recall: 24 h	9 components scored—food groups and nutrients; each component scored ≤10–20 proportionally based on meeting recommendations in servings, intakes or % of TEI; summed Final score, 0–100, higher score indicating healthier diet; continuous scoring only	Very low	Low	Low
Healthy Eating Index—2010: 2013 [84]	Adherence to 2010 dietary guidelines for Americans	Diversity (implicit) Favorable dietary pattern (implicit) Moderation (implicit)	United States (national)	2–19 y	Quantitative 24HR • Respondent: not stated • Recall: 24 h	12 components scored—food groups and nutrients; each component scored proportionally based on meeting recommendations in intakes or % of TEI; summed Final score, 0–100, higher score indicating healthier diet; continuous scoring only	Very low	Low	Low
Adapted Healthy Eating Index—2010: 2019 [85]	Adherence to Brazilian food habits and the Brazilian Ministry of Health dietary recommendations	Diversity (implicit) Favorable dietary pattern (implicit) Moderation (implicit)	Brazil (urban)	8–12 y	Quantitative 24HR • Respondent: child • Recall: 24 h	12 components scored—food groups and nutrients; each component scored proportionally based on meeting recommendations in intakes or % of TEI; summed Final score, 0–100, higher score indicating healthier diet; continuous scoring only	Very low	Low	Low
Healthy Eating Index—2015-TUBER: 2022 [86]	Adherence to Turkey dietary guidelines	Information not provided	Turkey (Kayseri—urban)	6–17 y	Food diary • Respondent: not stated • Recall: 3 d measured	Calculation details not provided. Final score 0–100, higher score indicating healthier diet; 3 categories: ≤50 “poor diet,” 51–80 “diet needs improvement,” >80 “good diet”	—	—	Low
Children’s Diet Quality Index (C-DQI): 2004 [87]	Adherence to 1998 Food Guide Pyramid for 2–6 y olds	Diversity (implicit) Favorable dietary pattern (implicit) Moderation (implicit) Nutrient adequacy (explicit)	United States (national)	2–5 y	Quantitative 24HR • Respondent: caregiver • Recall: 24 h	12 components scored—food groups and nutrients; each component scored proportionally based on meeting recommendations in intakes or % of TEI; summed Final score, 0–100, higher score indicating healthier diet; continuous scoring only	Very low	Low	Low
Revised Children’s Diet Quality Index (RC-DQI): 2006 [88]	Adherence to 1998 Food Guide Pyramid for 2–6 y olds	Diversity (implicit) Favorable dietary pattern (implicit) Moderation (implicit) Nutrient adequacy (explicit) Other: sedentary behavior	United States (national)	2–5 y	Quantitative 24HR • Respondent: caregiver • Recall: 24 h	13 components scored—food groups, behavior and nutrients; each component scored proportionally based on meeting recommendations in intakes or % of TEI; summed Final score, 0–95, higher score indicating healthier diet; continuous scoring only	Very low	Low	Low
Modified Revised Children’s Diet Quality Index (M-RDQI): 2018 [89]	Adherence to 2005 dietary guidelines for Americans	Diversity (implicit) Favorable dietary pattern (implicit) Moderation (implicit)	Iran (Tehran)	13–15 y	Semiquantitative FFQ • Respondent: child • Recall: 4 wk	13 components scored—food groups and nutrients; each component scored proportionally based on meeting recommendations in intakes, EAR or % of TEI; summed Final score, 0–90, higher score indicating healthier diet; continuous scoring only	Very low	Low	Low
Variety Index for Toddlers (VIT): 1997 [90]	Adherence to United States Food Pyramid Guidelines	Diversity (explicit)	United States (setting not stated)	2–3 y	Quantitative 24HR • Respondent: caregiver • Recall: 24 h	5 components scored—food groups; each component scored 0–5 or 0–10 based on ratio of recommended serving quantities to consumed quantities and truncated at 1; averaged Final score, 0–1, higher score indicating healthier diet; continuous scoring only	Low	High	High
Food Variety Index for Children (VIC): 1999 [91]	Adherence to United States Food Pyramid Guidelines	Diversity (explicit)	United States (Knoxville, TN—urban)	2–5 y	Quantitative 24HR • Respondent: caregiver • Recall: 24 h	5 components scored—food groups; each component scored 0–5 or 0–10 based on ratio of recommended serving quantities to consumed quantities and truncated at 1; averaged Final score, 0–1, higher score indicating healthier diet; continuous scoring only	Low	High	Low
Healthy Plate Variety Score: 2015 [92]	Adherence to United States Health Eating Guidelines	Diversity (explicit)	United Kingdom, France, Greece, Portugal (setting not stated)	2–4 y	Qualitative FFQ • Respondent: caregiver • Recall: not stated	5 components scored—food groups; each component scored based on ratio of recommended servings to consumed servings and truncated at 1; summed Final score, 0–5, higher score indicating healthier diet; continuous scoring only	Medium	High	High
Healthy Dietary Variety Index: 2019 [93]	Adherence to Portugal healthy eating guidelines	Diversity (explicit)	Portugal (Porto—urban)	4 y	Qualitative FFQ • Respondent: caregiver • Recall: 6 mo	5 components scored—food groups; each component scored based on ratio of recommended servings to consumed servings and truncated at 1; averaged Final score, 0–1, higher score indicating healthier diet; continuous scoring only	Medium	High	Low
Nutrient Rich Foods Index (NRF 9.3): 2009 [94]	Adherence to dietary guidelines for Americans	Nutrient adequacy (explicit)	Not stated	4–19 y	Quantitative 24HR • Respondent: not stated • Recall: 24 h	12 components—nutrients; each scored based on percentage of reference intake met; summed Continuous score, higher score indicating healthier diet	Very low	High	Low
Grain, Fruit, Vegetables, Dairy and Meat Variety Score (GFVDM): 2004 [95]	Adherence to 1992 United States Food Guide Pyramid food groups	Diversity (explicit)	United States (Cincinnati—urban)	11–12 y	Quantitative 24HR • Respondent: child • Recall: 24 h (14 d measured)	5 components—food groups; each component scored based on number of servings consumed Continuous score, higher score indicating healthier diet	Medium	High	Low
Grain, Fruit, Vegetables Variety Score (GFV): 2004 [95]	Adherence to 2000 United States dietary guidelines	Diversity (explicit)	United States (Cincinnati—urban)	11–12 y	Quantitative 24HR • Respondent: child • Recall: 24 h (14 d measured)	3 components—food groups; each component scored based on number of servings consumed Continuous score, higher score indicating healthier diet	Medium	High	Low
Dietary Approaches to Stop Hypertension (DASH) score: 2009 [96]	Adherence to DASH dietary pattern and dietary guidelines for Americans	Diversity (implicit) Favorable dietary pattern (implicit) Moderation (implicit)	United States (Ohio, Washington, South Carolina, Colorado, Hawaii, California)	10–19 y	Qualitative FFQ • Respondent: child • Recall: 7 d	8 components scored—food groups; each component scored 0–10 proportionally to whether number of servings consumed met recommended number; summed Final score, 0–80, higher score indicating healthier diet; continuous scoring only	Medium	Low	Low
Finnish Children Healthy Eating Index: 2014 [97]	Adherence to Nordic dietary guidelines	Diversity (implicit) Favorable dietary pattern (implicit) Moderation (implicit)	Finland (north and south)	3 and 6 y	Weighed food records • Respondent: caregiver • Recall: 3 d	5 components scored—food groups; each component scored 0–10 proportionally to whether quantities consumed per energy intake met recommendations; summed Final score, 0–41 for 3 y old and 0–42 for 6 y olds, higher score indicating healthier diet; continuous scoring only	Very low	Low	High
Nutrition Quality Index (NQI): 2001 [98]	Adherence to 2002 German, Austrian and Swiss dietary reference values	Nutrient adequacy (explicit)	Germany (setting not stated)	4–19 y	Quantitative 24HR • Respondent: not stated • Recall: 24 h	On the basis of intake of 35 nutrients across 3 groups (those with maximum requirements, those with minimum requirements, and those with equality requirement); scored 1–100 for each nutrient meeting % of requirement and then all 35 averaged Final score, 0–100, higher score indicating healthier diet; continuous scoring only	Very low	High	High
Dietary Guideline Index for Children and Adolescents (DGI CA): 2011 [99]	Adherence to 2003 Australian dietary guidelines for children and adolescents and 1998 Australian Guide to Healthy Eating	Diversity (implicit) Favorable dietary pattern (implicit) Moderation (implicit) Other: water consumption	Australia (national)	4–16 y	Quantitative 24HR • Respondent: child (9 y and older), caregiver (4–8 y) • Recall: 24 h	11 components scored—food groups; each component scored 0–5 or 0–10 proportionally to whether servings or % of TEI met recommendations; summed Final score, 0–100, higher score indicating healthier diet; continuous scoring only	Very low	Low	Low
Modified Dietary Guideline Index: 2014 [100]	Adherence to 2003 Australian dietary guidelines for children and adolescents	Diversity (implicit) Favorable dietary pattern (implicit) Moderation (implicit) Other: water consumption	Australia (urban and rural)	5–12 y	Qualitative FFQ • Respondent: caregiver • Recall: 4 wk	10 components scored—food groups; each component scored 0–10 proportionally based on servings/frequency of consumption per day; summed Final score, 0–100, higher score indicating healthier diet; continuous scoring only	Medium	Low	Low
Healthy Diet Score: 2010 [101]	Adherence to Australian dietary guidelines for children and adolescents	Diversity (implicit)	Australia (national)	10–14 y	Questionnaire • Respondent: not stated • Recall: not stated	4 components—food recommendations; each component scored 1 point if met; summed Final score, 1–5, higher score indicating healthier diet; continuous scoring only	High	High	Low
Unhealthy Diet Score: 2010 [101]	Adherence to Australian dietary guidelines for children and adolescents	Moderation (implicit)	Australia (national)	10–14 y	Questionnaire • Respondent: not stated • Recall: not stated	5 components—food groups; each component scored 1–6 based on frequency; summed Final score, 1–30, higher score indicating healthier diet; continuous scoring only	High	High	Low
DQI: 2012 [102]	Adherence to Australian Guide to Healthy Eating and nutrient reference values for Australia and New Zealand	Diversity (implicit) Favorable dietary pattern (implicit) Moderation (implicit) Nutrient adequacy (explicit)	Australia (national)	2–14 y	Semiquantitative FFQ • Respondent: caregiver (in consultation with child) • Recall: 12 mo	15 components—food groups and nutrients; each component scored ≤10 based on nutrient intake and servings; summed Final score, 20–150, higher score indicating healthier diet; continuous scoring only	Medium	Low	Low
Core Food Variety Score: 2012 [103]	Adherence to Australian Guide to Healthy Eating	Diversity (implicit) Favorable dietary pattern (implicit) Moderation (implicit)	Australia (Perth—urban)	2 y	Quantitative 24HR • Respondent: caregiver • Recall: 24 h	5 components—food groups; each component scored up based on consumption of foods within food group in previous 24h; summed Final score, 0–34, higher score indicating healthier diet; continuous scoring only	Medium	Low	Low
Short Food Frequency Questionnaire Diet Quality Index (sFFQ-DQI): 2018 [104]	Adherence to 2013 Australian dietary guidelines for children and adolescents	Diversity (implicit) Favorable dietary pattern (implicit) Moderation (implicit) Other: water consumption, sedentary behavior, breakfast consumption	Australia (setting not stated)	2–5 y	Qualitative FFQ • Respondent: caregiver • Recall: not stated	13 components—food groups and eating behavior; each component scored based on frequency of consumption in previous d/wk; summed Final score, 0–65, higher score indicating healthier diet; continuous scoring only	Medium	Low	Low
Diet Quality Index for Preschool Children (DQI-CH): 2010 [105]	Adherence to Flemish dietary guidelines	Diversity (explicit) Favorable dietary pattern (explicit) Moderation (explicit)	Belgium (Flanders)	2–6 y	Qualitative FFQ • Respondent: caregiver • Recall: 12 mo	4 components—dietary diversity (consumption of ≥1 serving of food per day from each of 8 recommended food groups), dietary quality (consumption of portions from 3 groups: preference, moderation, and low-nutritious, energy-dense), dietary equilibrium (disaggregation of adequacy and moderation of diet), meal index (frequency of breakfast, lunch and dinner across a week); scores of all 4 (based on quantities per day consumed) were summed and divided by 4 Final score, –25 to 100, higher score indicating healthier diet; continuous scoring only	Medium	Low	Low
Diet Quality Index for Adolescents (DQI-A): 2012, 2013 [106,107]	Adherence to Flemish dietary guidelines	Diversity (explicit) Favorable dietary pattern (explicit) Moderation (explicit)	Austria (Vienna), Belgium (Ghent), France (Lille), Germany (Dortmund), Greece (Athens), Hungary (Pecs), Italy (Rome), Spain (Zaragoza), Sweden (Stockholm)—urban	12.5–17.5 y	Quantitative 24HR • Respondent: child • Recall: 24 h	On the basis of,3 components, each scored separately—dietary diversity (based on food group consumption), dietary quality (based on food group consumption weighted for healthy or unhealthy), dietary equilibrium (scored based on how much servings sizes correspond to quantities recommended); all 3 component scores summed Final score, –33 to 100, higher score indicating healthier diet; continuous scoring only	Low	Low	High
Daily Diversity Index (DDI): 2008 [108]	Adherence to 2000 American Food Guide Pyramid	Diversity (implicit)	Belgium (Flemish regions)	10 y	Qualitative FFQ • Respondent: child • Recall: 7 d	5 components—food groups; each component scored based on daily consumption during the previous week 0–1; summed Final score, 0–5, higher score indicating healthier diet; continuous scoring only	Medium	High	Low
Índice de Alimentação do Escolar (ALES—School Child Diet Index): 2010 [109]	Adherence to Brazil national dietary guidelines	Diversity (implicit) Favorable dietary pattern (implicit) Moderation (implicit)	Brazil (Vitoria—urban)	7–10 y	Qualitative FFQ • Respondent: caregiver • Recall: not stated	16 components—food groups; each component scored based on frequency of consumption; summed Final score, –15 to 16, higher score indicating healthier diet; 3 food quality categories: <3, poor quality; between 3 ≥ and <6, average quality; ≥6, good quality	Medium	Low	Low
Chinese Healthy Eating Index: 2017 [110]	Adherence to 2016 dietary guidelines for China	Diversity (implicit) Favorable dietary pattern (implicit) Moderation (implicit)	China (national)	2–19 y	Quantitative 24HR • Respondent: not stated • Recall: 24 h	17 components—food groups and nutrients; each component scored proportionally based on adherence to recommendation total intake and intake per 1000 kcal, and intakes; summed Final score, 0–100, higher score indicating healthier diet; continuous scoring only	Very low	Low	Low
Australian Recommended Food Score for Preschoolers (ARFS-P): 2014 [111]	Adherence to Australian dietary guidelines	Diversity (implicit) Favorable dietary pattern (implicit) Moderation (implicit)	Australia (rural)	2–5 y	Qualitative FFQ • Respondent: caregiver • Recall: 6 mo	8 components—food groups; each component scored based on consumption frequency; summed Final score, 0–73, higher score indicating healthier diet; continuous scoring only	Medium	Low	Low
Healthy Dietary Score: 2017 [112]	Adherence to 2013 Australian dietary guidelines	Diversity (implicit) Favorable dietary pattern (implicit) Moderation (implicit)	Australia (national)	2–15 y	Qualitative 24HR • Respondent: child (10 y and older), caregiver (2–9 y) • Recall: 24 h	7 components—food groups; each component scored 0–2 based on consumption frequency; summed Final score, 0–14, higher score indicating healthier diet; continuous scoring only	Medium	Low	Low
Diet Quality Index for Indian Children (DQIIC): 2019 [113]	Adherence to dietary guidelines for Indian children	Diversity (implicit) Favorable dietary pattern (implicit) Moderation (implicit) Nutrient adequacy (explicit) Other: breakfast consumption, eating outside the home, eating packaged foods	India (Srinagar Garhwal, Uttarakhand— rural)	7–9 y	Quantitative 24HR • Respondent: child • Recall: 24 h	21 components—food groups and nutrients; each component scored based on intake quantities, servings or frequency of consumption/behavior; summed Final score, 0–90, higher score indicating healthier diet; 3 categories: 78–90: healthy diet, 73–77 moderately healthy diet, 0–72 unhealthy diet	Very low	Low	Low
Diet Risk Score: 2024 [114]	Adherence to Australian dietary guidelines for infants, toddlers and children	Moderation (implicit)	Australia (setting not stated)	3–5 y	Questionnaire • Respondent: caregiver • Recall: 7 d	9 components—food groups; each component scored 0–2 based on consumption frequency; summed Final score, 0–72, higher score indicating healthier diet; continuous scoring only	High	High	Low
Dietary Index for Child’s Eating (DICE): 2019 [115]	Adherence to New Zealand Food and Nutrition Guidelines for 2–18 y olds	Diversity (implicit) Favorable dietary pattern (implicit) Moderation (implicit) Other: regular meal and snack consumption	New Zealand (national)	2–8 y	Questionnaire • Respondent: caregiver • Recall: no stated	13 components—food groups and eating behaviors; each component scored; summed Final score, 0–100, higher score indicating healthier diet; continuous scoring only	High	Low	Low
DQI – Canada: 2020 [116]	Adherence to Canada’s Food Guide	Diversity (implicit) Favorable dietary pattern (implicit) Moderation (implicit)	Canada (Calgary and Edmontha, Alberta—urban)	3 y	Semiquantitative FFQ • Respondent: caregiver • Recall: 7 d	6 components—food groups; each component scored based on ratio of reported intake (grams per day) to recommended intake, truncated at 1; unhealthy food groups reverse scored; summed Final score, 0–6, higher score indicating healthier diet; continuous scoring only	Low	Low	Low
Adapted Chinese Diet Balance Index Revision (DBI-07): 2022 [117]	Adherence to Chinese dietary guidelines	Diversity (implicit) Favorable dietary pattern (implicit) Moderation (implicit)	China (urban)	9–10 y	Semiquantitative FFQ • Respondent: not stated • Recall: 7 d	12 components—food groups; “over intake” groups considered unhealthy and received positive points, whereas “under intake” groups considered healthy and received negative points; if intake exceeded 25 g, a score of 1 was given On the basis of, the scores evaluated, the intake of each food subgroup was divided into underintake or overintake as categorical variables, and dietary diversity was divided into adequate or inadequate. Three indicators of overall dietary quality were calculated. HBS were the sum of all the positive scores, indicating the overall overintake. LBS were the sum of the absolute values of all the negative scores, indicating the overall undertake. DQD was calculated by adding the absolute values of all the positive and negative scores, indicating the overall unbalanced intake	Low	Low	Low
Adapted Prime Diet Quality Score: 2024 [118]	Adherence to 2020 dietary guidelines for Americans	Diversity (implicit) Favorable dietary pattern (implicit) Moderation (implicit)	United States (Massachusetts)	3, 8, 13, and 18 y	Semiquantitative FFQ (3 y olds) • Respondent: caregiver • Recall: 1 mo Prime Screen Questionnaire (8 and 13 y olds) • Respondent: child (13 y), caregiver (8 y) • Recall: 1 mo Quantitative 24HR (18 y olds) • Respondent: child • Recall: 1 mo	10 adequacy components for healthy score and 5 moderation components for unhealthy score; each component scored 0–2 based on frequency of consumption per week or day depending on age. Healthy PDQS score 0–20, higher score indicating healthier diet; Unhealthy PDQS score 0–10, higher score indicating unhealthier diet; continuous score for both	Medium	Low	Low
7 food group Minimum Dietary Diversity: 2021 [119]	Reflection of mean probability of nutrient adequacy	Diversity (explicit) Nutrient adequacy (implicit)	Burkina Faso	2–4 y	Qualitative 24HR • Respondent: caregiver • Recall: 24 h	7 components—food groups; each component scored 0–1 based on consumption in previous day; summed Final score, 0–7, higher score indicating healthier diet; cut off of 4 or more food groups indicating dietary diversity	High	High	High
10 food group Minimum Dietary Diversity: 2021, 2024 [119,[120], [121], [122], [123]]	Reflection of mean probability of nutrient adequacy	Diversity (explicit) Nutrient adequacy (implicit)	Argentina, Bangladesh, Bolivia, Brazil, Bulgaria, Burkina Faso, China, Costa Rica, Ethiopia, India, Italy, Kenya, Lao PDR, Mexico, Mozambique, Nigeria, Romania, Tanzania, Uganda, Zambia	2–19 y	Qualitative 24HR • Respondent: child (16 y and older), caregiver (2–15 y) • Recall: 24 h	10 components—food groups; each component scored 0–1 based on consumption in previous day; summed Final score, 0–10, higher score indicating healthier diet; cut off of 4/5 or more food groups indicating dietary diversity (cut off dependent on context and age population)	High	High	High
Adolescent Adapted Global Diet Quality Score (A-GDQS): 2023 [124]	Literature on dietary contributors to nutrient intakes and noncommunicable disease risk globally	Diversity (implicit) Favorable dietary pattern (implicit) Moderation (implicit)	Burkina Faso, Ethiopia, Sudan and Tanzania (urban)	10–15 y	Qualitative FFQ • Respondent: child • Recall: 7 d	17 components—food groups; each component scored 0–4 based on servings per week; reverse scoring for unhealthy food groups; summed Final score 0–40, higher score indicating higher diet quality; 2 categories: poor diet quality (GDQS < 10), and low risk for poor diet quality (GDQS > 23)	High	Low	High
Food-based Diet Score: 2019 [125]	Adherence to Nordic nutrition recommendations and 2015 United States dietary guidelines for Americans	Diversity (implicit) Favorable dietary pattern (implicit) Moderation (implicit)	Finland (Turku)	2–19 y	Weighed food records • Respondent: caregiver and daycare/school personnel • Recall: 4 d	11 components—food groups; each component scored 0–3 points based on quartiles of consumption in grams/energy intake; unhealthy food groups reverse scored; summed Final score, 0–33, higher score indicating healthier diet; continuous scoring only	Very low	Low	High
Modified Diet Quality Index Score (DQIS): 2020 [126]	Adherence to United States dietary guidelines and WHO IYCF indicator guidance	Diversity (implicit) Favorable dietary pattern (implicit) Moderation (implicit)	United States (national)	2–4 y	Qualitative 24HR • Respondent: caregiver • Recall: 24 h	10 components—food groups; each component scored 0–2.5 or 0.5 points based on number of portions/quantities; summed Final score, 0–45, higher score indicating healthier diet; continuous scoring only	Low	Low	High
Alternative Healthy Eating Index (AHEI): 2016 [127]	Adherence to 2005 United States dietary guidelines	Diversity (implicit) Favorable dietary pattern (implicit) Moderation (implicit)	United States (14 states)	13–18 y	Semiquantitative FFQ • Respondent: child • Recall: not stated	8 components—food groups; each component scored 0–10 points based on grams or servings/day; summed Final score, 0–80, higher score indicating healthier diet; continuous scoring only	Low	Low	Low
Healthy Diet Score: 2020 [128]	Adherence to 2005 United States dietary guidelines	Diversity (implicit) Favorable dietary pattern (implicit) Moderation (implicit)	Peru (Lima—urban)	9–19 y	Qualitative FFQ • Respondent: child • Recall: 14 d	11 components—food groups; each component scored 0–4 points based on quartiles of frequency of consumption; reverse scoring for unhealthy food groups; summed Final score, 0–44, higher score indicating healthier diet; continuous scoring only	Medium	Low	Low
Adapted Alternative Healthy Eating Index (AHEI) 2010: 2020 [129]	Adherence to 2005 United States dietary guidelines	Diversity (implicit) Favorable dietary pattern (implicit) Moderation (implicit)	Colombia (national)	4–17 y	Quantitative 24HR • Respondent: not stated • Recall: 24 h	10 components—food groups and nutrients; each component scored 0–10 points based on servings/quantities/% of TEI; summed Final score, 0–100, higher score indicating healthier diet; continuous scoring only	Very low	Low	Low
Food Quality Score: 2021 [130]	Adherence to 2015 Dutch guidelines for a Healthy Diet	Diversity (implicit) Favorable dietary pattern (implicit) Moderation (implicit)	Iran (Tehran)	6 y	Semiquantitative FFQ • Respondent: caregiver • Recall: 4 wk	10 components—food groups; each component scored based on grams per day consumed compared with intake cutoffs; summed Final score, 0–18, higher score indicating healthier diet; continuous scoring only	Low	Low	Low
Adapted Index of Diet Quality: 2022 [131]	Adherence to Finnish national dietary guidelines	Diversity (implicit) Favorable dietary pattern (implicit) Moderation (implicit)	Finland (southwest of country)	2 and 5 y	Questionnaire • Respondent: caregiver • Recall: not stated	10 components—food groups; each component scored 0–1 based on adherence to recommendations; summed Final score, 0–10, higher score indicating healthier diet; continuous scoring only	High	Low	Low
Diet Quality Index (DQI-5): 2023 [132]	Adherence to Singapore dietary recommendations for children 3–6 y	Diversity (explicit) Favorable dietary pattern (explicit) Moderation (explicit)	Singapore (setting not stated)	5 y	Semiquantitative FFQ • Respondent: caregiver • Recall: not stated	12 components—food groups and nutrients; each component scored 0–10 proportionally based on servings, quantities or % of TEI; summed Final score, 0–110, higher score indicating healthier diet; cut off of 4 or more food groups indicating dietary diversity for Costa Rica and 5 of more for Mexico	Very low	Low	Low
Modified Global Dietary Recommendation (GDR) scores: 2023 [133]	Adherence to WHO Healthy Diet Fact Sheet 2018	Diversity (implicit) Favorable dietary pattern (implicit) Moderation (implicit)	China (Shandong province)	11–18 y	Qualitative 24HR • Respondent: child • Recall: 7 d	Calculation details not provided. Higher GDR score, higher GDR-Healthy score and lower GDR-Limit score indicate healthier diet	Medium	Low	High
Adapted Lifelines Diet Score (LLDS): 2021 [134]	Adherence to 2015 Dutch dietary guidelines	Diversity (implicit) Favorable dietary pattern (implicit) Moderation (implicit)	Netherlands (setting not stated)	3 y	Semiquantitative FFQ • Respondent: caregiver • Recall: 4 wk	8 components—food groups; each component scored 0–4 based on quintiles of consumption in g/1000 kcal (inverse scoring for unhealthy groups); summed Final score, 0–44, higher score indicating healthier diet; continuous scoring only	Very low	Low	Low
Dietary Diversity Score: 1996 [135]	Adherence to dietary guidelines for Americans	Diversity (explicit)	Gaza (urban and semirural)	12–13 and 17–18 y	Qualitative FFQ • Respondent: not stated • Recall: not stated	4 components—food groups; each component scored 1 if consumed; summed Final score, 0–4, higher score indicating healthier diet; continuous scoring only	High	High	Low
Adapted DQI: 2017 [136]	Adherence to Danish national dietary guidelines	Diversity (implicit) Favorable dietary pattern (implicit) Moderation (implicit)	Denmark (Copenhagen—urban)	2–6 y	Weighed food records • Respondent: caregiver • Recall: 4 d	6 components—food groups and nutrients; each component scored 0–1 based on ratio of reported intakes to recommended intakes (grams per day or % of TEI), truncated at 1; summed Final score, 0–6, higher score indicating healthier diet; continuous scoring only	Very low	Low	Low
Adapted Ideal Diet Score: 2017 [137]	Adherence to American Heart Association guidelines for children	Diversity (implicit) Favorable dietary pattern (implicit) Moderation (implicit)	Austria, France, Germany, Greece, Spain (urban)	12.5–17.5 y	Quantitative 24HR • Respondent: child • Recall: 24 h	5 components—food groups and nutrients; each component scored 0–1 based on meeting recommended intakes (quantities per day); summed Final score, 0–5, higher score indicating healthier diet; continuous scoring only	Very low	Low	High
Adapted Baltic Sea Diet Score (BSDS): 2017 [138]	Adherence to Baltic Sea Diet Pyramid	Diversity (implicit) Favorable dietary pattern (implicit) Moderation (implicit)	Finland (Kuopio—urban)	6–8 y	Weighed food records • Respondent: caregiver • Recall: 4 d measured	8 components—food groups and nutrients; each component scored proportionally 0–3 based on meeting recommended intakes (grams/day or % of TEI); summed Final score, 0–24, higher score indicating healthier diet; continuous scoring only	Very low	Low	Low
Adapted Dietary Guidelines for Americans Adherence Index (mDGAI): 2016 [139]	Adherence to dietary guidelines for Americans	Diversity (implicit) Favorable dietary pattern (implicit) Moderation (implicit)	Iran (Tehran—urban)	10–19 y	Semiquantitative FFQ • Respondent: child • Recall: 12 mo	19 components—food groups and nutrients; each component scored 0, 0.5 or 1 based on meeting recommended g/1000 kcal or % of TEI; summed Final score, 0–19, higher score indicating healthier diet; continuous scoring only	Very low	Low	Low
Food-based Diet Quality Score: 2016 [140]	Adherence to Japanese dietary guidelines	Diversity (implicit) Favorable dietary pattern (implicit) Moderation (implicit)	Japan (northern and western)	18 y	Questionnaire • Respondent: child • Recall: not stated	7 components—food groups and nutrients; each component scored proportionally 0–10 based on meeting recommended servings and energy/nutrient intakes; summed Final score, 0–70, higher score indicating healthier diet; continuous scoring only	Very low	Low	Low
A Priori Diet Quality Score (APDQS): 2016 [141]	Adherence to Mediterranean Diet Model	Diversity (implicit) Favorable dietary pattern (implicit) Moderation (implicit)	United States (Minnesota—urban)	15 y	Semiquantitative FFQ • Respondent: child • Recall: not stated	34 components—food groups and nutrients; each component scored proportionally 0–4 based on quartiles of intake (servings/day and mean daily nutrient intake); summed Final score, 0–136, higher score indicating healthier diet; continuous scoring only	Very low	Low	Low
Australian Child and Adolescent Recommended Food Score (ACARFS): 2012 [142]	Adherence to Dietary Guidelines for Children and Adolescents in Australia	Diversity (implicit) Other: water consumption	Australia (New South Wales)	6–14 y	Qualitative FFQ • Respondent: not stated • Recall: 6 mo	8 components—food groups; each component scored based on servings per week/day of FFQ items in the component; summed Final score, 0–73, higher score indicating healthier diet; continuous scoring only	Medium	High	Low
Adapted Nova ultraprocessed food (UPF) Score: 2023 [143]	Adherence to Nova classification system	Moderation (implicit)	Colombia (Medellín—urban)	14–20 y	Questionnaire • Respondent: not stated • Recall: 24 h	23 components—food groups; each component scored 1 point if consumed in the previous day; summed Final score, 0–23, higher score indicating unhealthier diet; continuous scoring only	High	High	High
Taiwanese Children Healthy Eating Index (TCHEI): 2024 [144]	Adherence to Taiwanese Food-Based Dietary Guideline	Diversity (implicit) Favorable dietary pattern (implicit) Moderation (implicit) Other: breakfast consumption	Taiwan (national)	2–6 y	Semiquantitative FFQ • Respondent: not stated • Recall: 4 wk	14 components scored—13 food groups and 1 behavior; each component scored 0–10 points based on frequency of consumption and proportion of intake Final score, 0–100, higher score indicating healthier diet, continuous scoring only	Very low	Low	Low
Diet quality index for adolescents adapted for Brazilians: 2020 (DQIA-BR) [145]	Adherence to Flemish dietary guidelines and Brazilian dietary guidelines	Diversity (explicit) Favorable dietary pattern (implicit) Moderation (explicit)	Brazil (national)	12–17 y	Quantitative 24HR • Respondent: child • Recall: 24 h	On the basis of3 components, each scored separately—dietary diversity (based on food group consumption), dietary quality (based on food group consumption weighted for healthy or unhealthy), dietary equilibrium (scored based on how much servings sizes correspond to quantities recommended); all 3 component scores summed Final score, –33–100, higher score indicating healthier diet; continuous scoring only	Very low	Low	Low
Adapted Japanese Food Guide Spinning Top score: 2020 [146]	Adherence to Japanese Spinning Top Guide	Diversity (implicit) Favorable dietary pattern (implicit) Moderation (implicit) Other: energy intake	Japan (Tokyo)	4–6 y	—	7 components—food groups and energy intake; each scored based on recommended servings or intake per day Final score, 0–70, higher score indicating healthier diet, continuous scoring only	Very low	Low	Low

Abbreviations: DQD, diet quality distance; FFQ, food frequency questionnaires; HBS, higher bound scores; LBS, lower bound scores; 24HR, 24-hour recall; TEI, total energy intake; EAR, estimated average requirement.

Eleven metrics (8.7%) reflected adherence to a national or localized dietary pattern, 12 metrics (9.4%) were based on global dietary guidance, 4 metrics (3.1%) aligned with regional dietary guidelines, and 2 metrics (1.6%) considered global evidence on foods associated with health risks or benefits. Most metrics aimed to assess multiple subconstructs of a healthy diet within a composite score, with diversity (*n =* 119, 93.7%) and moderation (*n =* 105, 82.7%) being the most widely included. Over 80% of metrics combined the subconstructs of diversity and moderation into a composite measure, representing a favorable dietary pattern. Other subconstructs of a healthy diet, such as nutrient adequacy (*n =* 10, 7.9%) and macronutrient balance (*n =* 1, 0.8%), were less frequently addressed. Twenty-six metrics (20.5%) measured additional subconstructs indirectly related to diet, including behaviors that could influence the healthiness of a child or adolescent diets, such as breakfast consumption (*n =* 8, 6.3%) and family meal participation (*n =* 4, 3.1%), as well as lifestyle behaviors such as sedentary activity (*n =* 5, 3.9%) and physical activity (*n =* 4, 3.1%). For most metrics (*n =* 98, 77.2%), the subconstructs reflected were implicitly identified by the researchers and only 29 metrics explicitly noted purposive measurement of their subconstructs of a healthy diet.

Over one-third of metrics (*n =* 48, 37.8%) had been developed or adapted among preschool age children (2–4 y), 55.9% (*n =* 71) among primary school children (5–9 y), 66.9% (*n =* 85) among younger adolescents (10–14 y), and 48.0% (*n =* 61) among older adolescents (15–19 y).

### Dietary data collection methods and respondents

A range of dietary assessment methods was used to collect data for the identified metrics. The most common method was quantitative 24-h dietary recalls with portion size estimation (*n =* 46, 36.2%), followed by nonquantitative food frequency questionnaires (FFQ) (only frequency of consumption collected) (*n =* 29, 22.8%) and semiquantitative FFQ (additionally including portion size estimation) (*n =* 29, 22.8%). A variety of recall periods were noted across metrics utilizing FFQ, ranging from 7 d to 12 mo. Eleven metrics (8.7%) used metric-specific diet history questionnaires. Just over one-third of the metrics (*n =* 46, 36.2%) relied on the child’s primary caregiver as the respondent, whereas 42.5% (*n =* 54) used the child or adolescent as the respondent (with the remaining metrics not specifying the respondent). Of the 46 metrics where the primary caregiver served as the respondent, 78.3% (*n =* 36) of these were for children aged ≤10 y. Ten metrics involved the caregiver providing dietary intake information for younger children, with the child being the respondent once they reached a certain age. Across these 10 metrics, the age cutoff for when caregivers compared with children and adolescents served as the respondent ranged from 7 to 16 y, with a median of 10 y.

### Suitability of identified metrics for global monitoring

The feasibility of computation and interpretation varied among the identified healthy diet metrics for children and adolescents (Table 4). Overall, for ease of computation, only 35 (27.6%) and 22 metrics (17.3%) were rated as medium or high, respectively. More than half (*n =* 70, 55.1%) required quantitative dietary intake data, such as the grams of foods or beverages consumed or energy/nutrient intakes. Most metrics (*n =* 103, 81.1%) were rated low for ease of interpretation. This was primarily due to the inclusion of both the subconstructs of diversity and moderation within 1 composite metric. Metrics aggregated across subconstructs do not allow discernment as to whether the directionality of scoring was related to high diversity or low consumption of foods/nutrients to moderate. For example, a low overall score could result from low dietary diversity combined with low consumption of foods to be moderated, or from high dietary diversity offset by high intake of foods to be moderated. Adaptability of the metrics was generally low, with only 28 metrics (22.0%) rated as high, either being based on regional or global dietary guidance or having been developed or adapted for use in >1 country.

Of the 127 metrics, only 5 were identified as suitable for high-frequency global monitoring based on their feasibility and adaptability (Table 5). All 5 were determined to have low or medium interviewer and respondent burden according to discussions with child and adolescent diet assessment experts. These were: *1*) the Individual Dietary Diversity Score (IDDS) [58], a metric measuring diversity of diets based on a simple count of consumption of 9 food groups across the previous day (scored as a discrete count); *2*) 7 food group Minimum Dietary Diversity (MDD)-7 [119], an adapted version of a WHO/UNICEF indicator for children aged 6–23 mo [147] measuring diversity of diets across the previous day based on a 7-point food group diversity score (FGDS) (dichotomously scored with a cutoff of ≥4 food groups); *3*) 10 food group MDD-10 [119,[120], [121], [122], [123]], an adapted version of a FAO indicator for women aged 15–49 y [148] measuring diversity of diets across the previous day based on a 10-point FGDS (dichotomously scored with a cutoff of ≥4 or 5 food groups based on the country and age subgroups); *4*) the Healthy Plate Variety Score [92], a metric measuring diversity of diets based on daily frequency of 5 food groups (continuously scored); and *5*) the Adapted Nova ultraprocessed food (UPF) score [143], an adapted version of a 2021 indicator [149] measuring the moderation of diets based on the consumption of 23 UPF subgroups in the previous day (continuously scored).

**TABLE 5:** Healthy diet metrics identified as suitable for global monitoring among children and adolescents.

Metric	Construction	Scoring
Individual Dietary Diversity Score [58]	10 components—food groups; each component receiving 1 point if consumed in previous day; summed Food groups: *1)* cereals and tubers; *2)* meat, poultry and fish; *3)* dairy; *4)* eggs; *5)* pulses and nuts; *6)* vitamin A–rich fruits and vegetables; *7)* other fruit; *8)* other vegetables; and *9)* oils and fats. Excluded a 10th food group of “other foods.”	Final score: 0–9, higher score indicating healthier diet; continuous scoring only
7 food group Minimum Dietary Diversity [119]	7 components—food groups; each component scored 0–1 based on consumption in previous day; summed Food groups: *1)* grains, roots, and tubers; *2)* legumes and nuts; *3)* dairy products; *4)* flesh foods; *5)* eggs; *6)* vitamin A–rich fruits and vegetables; and *7)* other fruits and vegetables	Final score: 0–7, higher score indicating healthier diet Cut off of ≥4 food groups indicating adequate dietary diversity
10 food group Minimum Dietary Diversity [119,[120], [121], [122], [123]]	10 components—food groups; each component scored 0–1 based on consumption in previous day; summed Food groups: *1)* grains, white roots and tubers, and plantains; *2)* pulses; *3)* nuts and seeds; *4)* dairy; *5)* flesh foods; *6)* eggs; *7)* dark-green leafy vegetables; *8)* vitamin A–rich fruits and vegetables; *9)* other vegetables; and *10)* other fruits	Final score: 0–10, higher score indicating healthier diet Cut off of ≥4 or ≥5 food groups indicating adequate dietary diversity, depending on context
Healthy Plate Variety Score [92]	5 components scored—food groups; each component scored based on ratio of recommended servings to consumed servings and truncated at 1; summed Food groups: *1)* starchy foods (including potatoes); *2)* fruits; *3)* vegetables; *4)* meat, fish, and alternatives; and *5)* dairy foods	Final score; 0–5, higher score indicating healthier diet; continuous scoring only
Adapted Nova UPF score [143]	23 components—UPF subgroups; each component scored 1 point if consumed in the previous day; summed UPF subgroups: *1)* regular or noncaloric, light, or zero calorie sodas; *2)* juice in a box or in a bottle; *3)* powdered mixes to prepare soft drinks; *4)* powdered mixes for chocolate drinks (powdered and ready-to-drink); *5)* tea drinks (powdered and ready-to-drink); *6)* fruit-flavored milk drinks; *7)* sausage, hamburger meat, or chicken nuggets; *8)* ham or mortadella; *9)* sliced bread, hot dog bun, or hamburger bun; *10)* table and cooking margarines; *11)* frozen French fries or fast-food-chain fries; *12)* ketchup, mayonnaise, or mustard sauce; *13)* salad dressings or vinaigrettes (ready-to-eat) *14)* instant noodles or packaged soups; *15)* frozen or fast-food restaurant pizza; *16)* lasagna or other frozen meals to heat or prepare at home; *17)* salted packaged products (chips or crackers); *18)* sweet biscuits with or w*ithout filling; 19) cake or packaged cake;* 20) cereal bars; *21)* ice cream or frozen popsicles; *22)* chocolates and chocolate bonbons; *23)* commercial breakfast cereal.	Final score: 0–23, higher score indicating unhealthier diet; continuous scoring only

Abbreviation: UPF, ultraprocessed food.

### Validity and reliability of metrics suitable for global monitoring

A total of 21 papers were identified with results among children and adolescents aged 2–19 y pertaining to the validity and reliability of the 4 of 5 metrics identified as suitable for global monitoring. Thirteen papers provided results for MDD-10 (8 with positive and 5 with neutral study quality), 5 papers pertained to the MDD-7 (3 with positive and 2 with neutral study quality), 3 papers pertained to the IDDS developed by Kennedy et al. [58] (all with positive study quality), and 1 paper pertained to the Adapted Nova UPF score (neutral study quality) (Table 6). No papers containing validation or reliability results were identified for the Healthy Plate Variety Score. Validation and reliability evidence were primarily for construct validity, cross-context equivalence, and predictive capacity for health or nutrition outcomes; no results on test-retest reliability or sensitivity to change over time were identified.

**TABLE 6:** Validity of healthy diet metrics suitable for global monitoring among children and adolescents.

Metric	Study	Setting and sample	Dietary assessment methods	Study quality^1^	Validation type	Results
10 food group MDD	Arimond et al., 2021 [123]	- China and Mexico (national) - *n =* 2306 (China); 6444 (Mexico) - 2–19 y; boys and girls - Data: 2011 CHNS and Mexican National Health and Nutrition Survey (ENSANUT-2012)	Quantitative 24HR • Respondent: not stated • Recall: 24 h	+	Construct Cross-context equivalence	Construct validity: in both Mexico and China, a positive and significant correlation was found between the 10-point food group diversity score and the energy-adjusted MPA of 11 micronutrients among children aged 2–4 (*Ρ* = 0.43 and 0.53) and 5–9 y (*Ρ* = 0.42 and 0.49), as well as among boys and girls aged 10–14 (*Ρ* = 0.33–0.41 and *Ρ* = 0.40–0.41) and 15-19 y (*Ρ* = 0.20–0.25 and *Ρ* = 0.38–0.53). The correlations were generally stronger in girls compared with boys and in the younger age groups. Cross-context equivalence: in Mexico, the best balance between sensitivity and specificity to predict an MPA >0.60 was achieved with a cutoff of ≥5 food groups for participants aged 10–14 and 15–19, and a cutoff of ≥4 food groups for children aged 2–4 and 5–9 y. In China, a ≥5 food group cutoff yielded the best balance for children aged 2–4 and 5–9 y, whereas a cutoff of ≥6 food groups demonstrated better sensitivity and specificity for adolescents aged 10–14 and 15–19 y.
10 food group MDD	Diop et al., 2021 [119]	- Burkina Faso (Boucle de Mouhoun, Centre-Ouest, and Haut-Bassins) - *n =* 1066 - 2–4 y; boys and girls - Data: Primary data collection; household survey	Quantitative 24HR • Respondent: caregiver • Recall: 24 h	+	Construct Cross-context equivalence	Construct: higher 10-point food group diversity score was positively and significantly (*P <* 0.001) correlated with a higher PA of 11 micronutrients (*Ρ* = 0.18–0.51) and energy-adjusted MPA (*Ρ* = 0.40). Cross-context equivalence: a cutoff of ≥4 food groups best predicted an MPA >0.75, providing the best balance across sensitivity, specificity, and proportion of correct classification.
10 food group MDD	Hanley-Cook et al., 2024 [122]	- Low income—Burkina Faso, Ethiopia, Mozambique, Uganda, Zambia; lower-middle income—Bangladesh, Bolivia, India, Kenya, Lao PDR, Nigeria, Tanzania; upper-middle income—Argentina, Brazil, Bulgaria, Mexico; high income—Italy, Romania - *n =* 75,480 - 10–19 y; boys and girls - Data: open access data on the FAO/WHO GIFT platform	Quantitative 24HR • Respondent: not stated • Recall: 24 h	+	Construct Cross-context equivalence	Construct: one-point increases in the 10-point food group diversity score was positively and significantly associated with 5.1 percentage points increment in MAR among boys and girls (95% CI: 5.0, 5.2). Correlations between the 10-point food group diversity score and MAR were *Ρ* = 0.36 and *Ρ* = 0.34 among adolescent boys and girls, respectively. Cross-context equivalence: a cutoff of ≥5 food groups best predicted the MAR >0.60 and provided the best balance across sensitivity, specificity, and proportion of correct classification among boys and girls from upper-middle and high-income countries. A cutoff of ≥4 food groups provided the best balance among girls in low-income countries and across both sexes in lower-middle income countries.
10 food group MDD	Monge-Rojas et al., 2024 [121]	- Costa Rica and Mexico (urban and rural) - *n =* 818 (Costa Rica); 1202 (Mexico) - 13–18 y; boys and girls - Data: 2016 Mexican Halfway National Health and Nutrition Survey (ENSANUT MC 2016)	Costa Rica Weighed food records • Respondent: child • Recall: 3 d Mexico Quantitative 24HR • Respondent: child • Recall: 24 h	+	Cross-context equivalence	Cross-context equivalence: a cutoff of ≥4 food groups of 10 in Costa Rica provided the best balance of sensitivity, specificity, and proportion of correct classification (71%, 64%, 70%, respectively) for an MPA >0.70, whereas a this was achieved by a cutoff of ≥5 food groups in Mexico (60%, 65%, and 64%, respectively).
10 food group MDD	Rodriguez-Ramirez et al., 2021 [120]	- Mexico (national) - *n =* 4806 - 5–19 y; boys and girls - Data: Mexican National Health and Nutrition Survey (ENSANUT-2012)	Quantitative 24HR • Respondent: child (≥16 y); caregiver assisted <16 y) • Recall: 24 h	+	Construct Cross-context equivalence	Construct: for both 5–11 y olds and 12–19 y olds, children who met ≥5 out of 10 food group cutoff had higher intakes of 11 micronutrients as compared with children who were below this cutoff (*P <* 0.05). Cross-context equivalence: a cutoff of ≥5 food groups best predicted a MPA >0.60, providing balance across sensitivity and specificity among both 5–11 y olds (60.5% and 72.8%, respectively) and 12–19 y olds (70.0% and 59.4%, respectively).
10 food group MDD	Wiafe et al., 2023 [150]	- Ghana (Oborgu and Banka) - *n =* 137 - 10–14 y; boys and girls - Data: primary data collection; household survey	Quantitative 24HR • Respondent: not stated • Recall: 24 h	○	Construct	Construct: controlling for adolescent age and sex, increments in the 10-point food group diversity score were positively and significantly correlated with intakes of 5 of 11 micronutrients: vitamin A (*r* = 0.169, *P <* 0.05), niacin (*r* = 0.186, *P <* 0.05), vitamin B6 (*r* = 0.191, *P <* 0.05), iron (*r* = 0.173, *P <* 0.05), and zinc (*r* = 0.175, *P <* 0.05). Nonsignificant correlations were reported for: thiamine (*r* = 0.126), folate (*r* =0.151), vitamin C (*r* = 0.143), calcium (*r* =0.046), sodium (*r* = 0.130) and riboflavin (*r* = –0.023).
10 food group MDD	de Castro-Mendes et al., 2021 [151]	- Portugal (Porto) - *n =* 590 - 7–12 y; boys and girls - Data: primary data collection; school-based survey	Quantitative 24HR • Respondent: child • Recall: 24 h	+	Predictive	Predictive—respiratory outcomes: children who consumed ≥5 of 10 food groups had lower odds of high levels of fractional exhaled nitric oxide (OR = 0.46, 95% CI: 0.24, 0.89). No significant associations were found between MDD-10 and positive bronchodilation or asthma. Children who achieved MDD-10 were more likely to report cough in the previous 12 mo. No significant associations were found between MDD-10 and lung function.
10 food group MDD	Getacher et al., 2023 [152]	- Ethiopia (Debre Berhan City) - *n =* 742 - 10–19 y; boys and girls - Data: primary data collection; school-based survey	Qualitative 24HR • Respondent: child • Recall: 24 h	+	Predictive	Predictive—anthropometric outcomes: 31.4% (*n =* 233) of adolescents consumed ≥5 of 10 food groups. Adolescents who failed to meet MDD-10 were more likely to be thin (AOR = 2.29, 95% CI: 1.27, 4.14) and also more likely to be overweight or obese (AOR = 5.26, 95% CI: 1.88, 14.671) compared with adolescents who consumed ≥ 5 food groups.
10 food group MDD	Islam et al., 2023 [153]	- Bangladesh (Matlab) - *n =* 2253 - Mean age: 15.0 ± 0.1 y; boys and girls - Data: follow-up of MINIMat cohort	Qualitative 24HR • Respondent: child • Recall: 24 h	○	Predictive	Predictive—cardiometabolic risk outcomes: Developed quartiles of diet quality based on 10-point food group score. Adolescents in the second (FGDS = 5) and top (FGDS ≥7) quartiles had 0.9 (95% CI: 0.1, 1.7, *P =* 0.029) and 1.4 (95% CI: 0.4, 2.4, *P =* 0.008) mm of Hg higher systolic blood pressure, respectively, compared with adolescents in the bottom quartile (FGDS ≤4). Compared with the boys in the bottom quartile, waist circumference was 1.9% (95% CI: 0.01%, 3.7%, *P =* 0.048) higher among the boys in the top quartile; no relationship between FGDS and waist circumference among girls was observed. Boys in the third quartile (DDS = 6) had 0.045 mmol/L (95% CI: 0.01, 0.08, *P =* 0.014) higher HDL than the boys in the bottom quartile. These associations were not observed among girls. No associations between FGDS quartiles and total cholesterol, triglyceride, LDL, or insulin resistance from Homeostasis Model Assessment were noted.
10 food group MDD	Mank et al., 2022 [154]	- Burkina Faso (Ouagadougou) - *n =* 1059 - 11–15 y; boys and girls - Data: primary data collection; school-based survey	Qualitative 24HR • Respondent: child • Recall: 24 h	○	Predictive	Predictive—anthropometric outcomes and anemia status: 37% of the adolescents consumed ≥5 of 10 food groups. Adolescents achieved MDD-10 were more likely to be thin (RR = 1.36, 95% CI: 1.06, 1.76, *P* < 0.05). There was no statistical difference between being overweight/obese or anemia status and achievement of MDD-10.
10 food group MDD	Mersha et al., 2021 [155]	- Ethiopia (Mirab-Armachiho District) - *n =* 706 - 10–19 y, girls only - Data: primary data collection; school-based survey	Qualitative 24HR • Respondent: child • Recall: 24 h	○	Predictive	Predictive—anthropometric outcomes: 55.9% of adolescent girls had consumed ≥5 of 10 food groups. The odds of being thin were 2.46 times (AOR = 2.46, 95% CI: 1.45, 4.20) higher among adolescents failing to achieve MDD-10 compared with reaching MDD-10. The odds of being stunted were 8 times (AOR = 8.07, 95% CI: 4.02, 16.20) higher among adolescents with not achieving MDD-10 compared with those with reaching MDD-10.
10 food group MDD	Partap et al., 2023 [156]	- Ethiopia (Addis Ababa), Sudan (Khartoum) and Tanzania (Dar es Salaam) - *n =* 3558 (*n =* 1200 Ethiopia, *n =* 1099 Sudan, *n =* 1255 Tanzania) - 10–14 y; boys and girls - Data: primary data collection; school-based survey	Qualitative 24HR • Respondent: child • Recall: 24 h	+	Predictive	Predictive—anemia status: 37.0% (*n =* 444), 18.5% (*n =* 203), and 6.3% (*n =* 79) of adolescents in Ethiopia, Sudan and Tanzania, respectively, consumed ≥5 of 10 food groups. Across the full sample, there was no association between achieving MDD-10 and any anemia (RR = 0.93, 95% CI: 0.81, 1.07, *P =* 0.314) or moderate/severe anemia (RR = 0.90, 95% CI: 0.55, 1.49, *P =* 0.691).
10 food group MDD	Wemakor et al., 2023 [157]	- Ghana (Kumbungu District) - *n =* 370 - 10–19 y; girls only - Data: primary data collection; household survey	Qualitative 24HR • Respondent: child • Recall: 24 h	○	Predictive	Predictive—anemia status: 57.3% (*n =* 212) of adolescent girls had consumed ≥5 of 10 food groups. 73.6% of those achieving MDD were anemic, as compared with 75.9% who did not; no association was found between achieving MDD-10 and anemia status (χ^2^ = 0.3, *P =* 0.605).
7 food group MDD	Diop et al., 2021 [119]	- Burkina Faso (Boucle de Mouhoun, Centre-Ouest, and Haut-Bassins) - *n =* 1066 - 2–4 y; boys and girls - Data: primary data collection; household survey	Quantitative 24HR • Respondent: caregiver • Recall: 24 h	+	Construct Cross-context equivalence	Construct: higher 7-point food group diversity score was positively and significantly (*P <* 0.001) correlated with a higher PA of 11 micronutrients (ρ = 0.17–0.45) and energy-adjusted MPA (ρ = 0.31). Cross-context equivalence: a cutoff of ≥4 food groups best predicted an MPA >0.75, providing the best balance across sensitivity, specificity, and proportion of correct classification.
7 food group MDD	Aboagye et al., 2022 [158]	- Ghana (South Tongu District) - *n =* 423 - 6–12 y; boys and girls - Data: primary data collection, school-based survey	Qualitative 24HR • Respondent: child • Recall: 24 h	+	Predictive	Predictive—anthropometric outcomes: 32.9% of children had consumed ≥4 of 7 food groups. The odds of being thin were 1.65 times (AOR = 1.65, 95% CI: 1.02, 2.67) higher among children achieving MDD-7 compared with those who did not achieve MDD-7.
7 food group MDD	Motadi et al., 2023 [159]	- South Africa (Vhembe district, Limpopo province) - *n =* 273 - 3–4 y; boys and girls - Data: primary data collection, school-based survey	Quantitative 24HR • Respondent: caregiver • Recall: 24 h	○	Predictive	Predictive—anthropometric outcomes: 59.6% and 73.5% of children aged 3 and 4 y, respectively, had consumed ≥4 of 7 food groups. Children who consumed <4 food groups had a significantly higher risk of being underweight (AOR = 0.25, 95 % CI: 0.08, 0.75) and stunted (AOR = 0.32, 95 % CI: 0.14, 0.74), as compared with those who consumed ≥4 food groups. Weight for height was not associated with MDD-7 (unadjusted; OR = 0.65, 95% CI: 0.43, 1.19; AOR = 0.33, 95 % CI: 0.32, 1.13).
7 food group MDD	Yazew, 2022 [160]	- Ethiopia (Oromia) - *n =* 500 - 3–5 y; boys and girls - Data: primary data collection; household survey	Qualitative 24HR • Respondent: caregiver • Recall: 24 h	○	Predictive	Predictive—anthropometric outcomes: 47.8% of children had consumed ≥4 of 7 food groups. The odds of being wasted were 3.4 times (AOR = 3.4, 95% CI: 3.10, 7.87) higher among children not achieving MDD-7 compared with those who did achieve MDD-7.
7 food group MDD	Bliznashka et al., 2021 [161]	- Benin, Burundi, Cambodia, Cameroon, Chad, Congo, Haiti, Honduras, Jordan, Maldives, Rwanda, Senegal, Timor-Leste, Togo, Uganda (national) - *n =* 12126 - 3–5 y; boys and girls - Data: pooled analysis of 15 Demographic and Health Surveys	Qualitative 24HR • Respondent: not stated • Recall: 24 h	+	Predictive	Predictive—cognitive outcomes: 18.2% of children had consumed ≥4 of 7 food groups. Increases in child FGDS or meeting MDD-7 were not associated with overall, cognitive, socioemotional or physical development. However, higher FGDS and meeting MDD-7 were associated with lower likelihood of suboptimal literacy-numeracy development, but the magnitude of these associations was very small. Achieving MDD-7 was associated with mean difference 0.12 (95% CI: 0.01, 0.23) higher Early Childhood Development Index score.
IDDS	Mak et al., 2019 [162]	- Philippines (national) - *n =* 6460 - 6–12 y; boys and girls - Data: 2013 Philippine National Nutrition Survey (NNS)	Quantitative 24HR • Respondent: caregiver • Recall: 24 h	+	Construct	Construct: when IDDS was 4 of the 9 food groups, the MPA across 11 micronutrients was ∼35%, which increased to 60% when all 9 food groups were consumed. The probability of adequacy of all nutrients improved with increasing DDS, with the exception of calcium, folate, vitamin A and vitamin C, where the PA remained at 0.
IDDS	Torrico et al., 2021 [163]	- Philippines (national) - *n =* 7448 - 3–18 y; boys and girls - Data: 2013 Philippine National Nutrition Survey (NNS)	Quantitative 24HR • Respondent: not stated • Recall: 24 h	+	Construct	Construct: included the 10th food group (other foods) that was not included in Kennedy et al [58]. Higher diversity score across the 10 food groups was positively and significantly (*P <* 0.001) associated with a higher MAR across 7 nutrients (calcium, iron, vitamin A, vitamin C, thiamine, riboflavin and niacin) among children aged 3–5, 6–12, and 13–18 y (ρ = 0.27, 0.33, and 0.29, respectively). A IDDS of ≥6 food groups showed the highest sensitivity (74.2%) and specificity (44.6%) for achieving MAR >0.50, whereas an IDDS of ≥7 food groups had the highest sensitivity (54.9%) and specificity (67.6%) in achieving MAR >0.75.
IDDS	Dogui et al., 2021 [164]	- Tunisia (Tunis) - *n =* 1164 - 3–9 y; boys and girls - Primary data collection; school-based survey	Quantitative 24HR • Respondent: caregiver • Recall: 24 h	+	Predictive	Predictive—anthropometric outcomes: IDDS score of 0–9 food groups was significantly higher in nonoverweight children as compared with overweight children among those <6 y (6.87 ± 0.07 compared with 6.04 ± 0.26, *P =* 0.002) and those ≥6 y (6.82 ± 0.07 compared with 6.21 ± 0.18, *P =* 0.002). In crude analysis, a 1-point increase in IDDS score was found to be negatively associated with overweight (crude OR = 0.81, 95% CI: 0.72, 0.89). However, in adjusted analysis, the increase of the IDDS score was associated with an increased risk of overweight (adjusted OR = 1.37, 95% CI: 1.03, 1.82).
Adapted Nova UPF score	Correa-Madrid et al., 2024 [143]	- Colombia (Medellín) - *n =* 203 - 14–19 y; boys and girls - Primary data collection; household survey	Questionnaire • Respondent: not stated • Recall: 24 h	○	Construct	Construct: mean (SD) Nova UPF score was 4.5 (2.57), with a range of 0–12. A positive and significant correlation was found between Nova UPF score and the proportion contribution of UPF to total energy intake (ρ = 0.51, *P <* 0.001), and for each 1-point increase in Nova Score the proportion of total energy intake from UPF increased by 2.8% on average (95% CI: 2.1, 3.6, *P <* 0.001). A positive and significant association was found between Nova UPF score and intake of nutrients related to chronic disease. For each 1-point increase in Nova Score, there was an increase of 212 mg of sodium intake (95% CI: 163, 262, *P <* 0.001), and the proportion of total energy intake from total fat and saturated fat increased by 0.73 percentage points (95% CI: 0.3, 1.16, *P <* 0.001) and 0.32 percentage points (95% CI: 0.13, 0.51, *P <* 0.001), respectively.

Abbreviations: CI, confidence interval; CHNS, China Health and Nutrition Survey; ENSANUT, Encuesta Nacional de Salud y Nutrición; FGDS, Food Group Diversity Score; IDDS, Individual Dietary Diversity Score; MAR, mean adequacy ratio; MDD, minimum dietary diversity; MINIMat, Maternal and Infant Nutrition Interventions in Matlab; MPA, mean probability of adequacy; OR, odds ratio; UPF, ultraprocessed foods.

1 Study quality based on Quality Criteria Checklist [15]: +, positive; ○, neutral; no studies were rated as negative.

### 10 food groups MDD-10

The MDD-10 metric demonstrated relatively consistent construct validity across diverse settings, with positive associations reported between food group diversity and micronutrient adequacy among children and adolescents in China, Mexico, Burkina Faso, and across 18 countries globally [119,122,123]. In Mexico and China, these associations were generally stronger among younger children and girls [123].

The cross-context equivalence of the food group cutoff for MDD-10 has been explored across a range of countries and age groups, with a cutoff of ≥4 or ≥5 food groups of 10 performing most consistently [119,[121], [122], [123]].

The predictive capacity of the MDD-10 metric for nutrition/health outcomes varied across contexts. Not meeting the MDD-10 cutoff of ≥5 food groups was associated with undernutrition in some adolescent populations [155,152], whereas meeting this cutoff was associated with an increased risk of thinness in other adolescent populations [154]. No associations were found between MDD-10 and anemia status [154,156,157]. One study noted an association between MDD-10 and cardiometabolic markers, with higher MDD-10 scores correlating with higher waist circumference, blood pressure, and HDL [153], whereas another found a positive association between achieving MDD-10 and lower odds of respiratory inflammation, [151].

### 7 food groups MDD-7

Evidence on the construct validity of MDD-7 is limited. One study from Burkina Faso found moderate correlations between MDD-7 and micronutrient adequacy among children aged 2–4 y, with a cutoff of ≥4 food groups offering the best balance between sensitivity and specificity [119]. MDD-10 performed slightly better than MDD-7 in predicting micronutrient adequacy in this setting [58].

The predictive validity of the MDD-7 metric for nutrition and health outcomes varied across contexts and ages. Achieving a cutoff of ≥4 food groups was associated with both increased and decreased risk of undernutrition depending on the country and age group [[158], [159], [160]]. One multicountry study found no overall associations with child development outcomes, though small positive associations were reported for early literacy and socioemotional development [161].

### Individual Dietary Diversity Score

Two studies assessing the construct validity of the IDDS developed by Kennedy et al. [58] reported positive associations with micronutrient adequacy among children and adolescents in the Philippines [162,163]. Associations were consistent across age groups, though some nutrients (e.g., calcium, folate, vitamin A, and vitamin C) showed weaker relationships in 1 study [162,163].

Limited evidence exists on predictive capacity, with 1 study reporting a higher risk of overweight among 3–9 y olds with higher diversity scores in urban Tunisia a [164]. No studies assessing the cross-context equivalence of IDDS were identified.

### Adapted Nova UPF score

The construct validity of the Adapted Nova UPF score was supported by 1 study among Colombian adolescents, which found that higher scores were associated with increased energy intake from UPFs and elevated intakes of sodium, total fat, and saturated fat [143]. No studies were identified assessing the cross-context equivalence or predictive capacity of the Adapted Nova UPF score for nutrition or health outcomes.

## Discussion

Despite the global urgency to improve the diets of children and adolescents, data on the healthiness of their diets remains scarce [165,166]. This gap is partly due to the lack of universally recognized low-burden metrics for monitoring healthy diets within this age group. Previous systematic reviews, including Marshall et al. [7] and Dalwood et al. [8], have cataloged existing diet quality indices used for children and adolescents, identifying a large and growing number of metrics. These reviews, however, did not evaluate which metrics are suitable for global monitoring purposes—a critical need given growing international efforts to track progress on nutrition and health targets. Findings from this review build on this prior work by identifying 127 distinct healthy diet metrics and critically assessing their feasibility, adaptability, and evidence base for global monitoring among children and adolescents aged 2–19 y.

Only 5 healthy diet metrics designed to capture the healthiness of child and adolescent diets demonstrated sufficient feasibility and adaptability to be considered suitable for global monitoring. This reinforces the concern that, although many metrics exist, few are practical for wide-scale application across diverse contexts. Most remaining metrics relied on quantitative dietary intake data that require substantial time and resources for data collection and processing, measure multiple subconstructs of healthy diets that obscures clear understanding of dietary challenges across populations [167], or were developed based on national dietary guidelines, limiting cross-country comparability. The strength of evidence supporting construct validity and cross-context equivalence varied across the 5 suitable metrics, with some metrics more extensively studied than others. Prior research has also underscored limited evidence on the cross-context equivalence of healthy diet metrics in adult populations [167]. Other gaps exist for all metrics, specifically regarding their sensitivity to change and test-retest reliability, highlighting the need for further research to support their use for global monitoring.

Of the 5 metrics identified as suitable for global monitoring (MDD-10, MDD-7, IDDS, Adapted Nova UPF Score, Healthy Plate Variety Score), MDD-10 was found to be a low-burden metric with high ease of computation and interpretation, and has the greatest body of evidence supporting its construct validity and cross-context equivalence in over 20 countries across Africa, Asia, Europe and Latin America. A cutoff of ≥5 food groups provided optimal sensitivity and specificity in higher-income settings, whereas a cutoff of ≥4 food groups was more appropriate in lower-income contexts, effectively predicting the mean adequacy ratio or the mean probability of adequacy of essential nutrients across different populations [119,122,123]. More recent validation of MDD-10 among 4–15-y-old children and adolescents across 7 low- and middle-income countries found that the optimal cutoff for predicting micronutrient adequacy varied between 4 and 6 food groups by country, but a cutoff of ≥5 demonstrated the highest performance in pooled analyses among this age group [168]. This variation underscores that no single cutoff is universally optimal—an insight consistent with previous findings from MDD-W validation studies among women, where the most appropriate cutoff ranged from 4 to 6 depending on the population/dietary pattern [123,169]. Despite this, a global cutoff of ≥5 for use of MDD-10 among children and adolescents has been proposed to support standardized monitoring and cross-country comparison [122]. Both the construct validity and cross-context equivalence evidence for MDD-10 were predominantly assessed among children and adolescents aged 5–19 y, and further research among preschool children aged 2–4 y is needed. Although this review found that the predictive associations between MDD-10 and diverse health outcomes have been inconsistent across contexts, MDD-10 is designed to capture dietary diversity and nutrient adequacy, rather than to directly predict energy balance, iron status, or cardiometabolic outcomes. Thus, its utility for global monitoring lies in its strength as a validated metric for diet diversity and nutrient adequacy, not as a predictor of all nutritional status and health outcomes.

A limitation of the 5 metrics identified in this review is their narrow focus on dietary diversity. The predominant focus of healthy diet metrics on dietary diversity, which can serve as a proxy for micronutrient adequacy, has been noted in other reviews of diet indices for populations of all ages [170]. Although diversity is a critical aspect of a healthy diet, facilitating the micronutrient adequacy needed for growing children and adolescents, the limited attention to other important subconstructs, particularly moderation, is concerning. Moderation is particularly relevant for this population given the rising consumption of unhealthy foods and growing global burden of childhood obesity [171]. Among the 5 metrics identified as suitable for global monitoring, only the Adapted Nova UPF score measured moderation, specifically targeting the intake of UPF [172]. Although UPF are an important health concern, particularly due to their association with obesity [173,174], metabolic disorders [175], and inadequate nutrient intakes [176], this metric does not fully capture the broader spectrum of unhealthy foods that are prevalent in children and adolescents’ diets. Foods high in added sugars, unhealthy fats, and sodium, which may not be classified as ultraprocessed, still pose substantial risks to children and adolescents’ health and nutritional status [177]. Therefore, although the Nova UPF score captures 1 aspect of moderation, it does not fully quantify all dietary components that should be limited. Additionally, some evidence suggests that not all UPF have the same impact on health outcomes, with certain foods, such as processed meats and sugar-sweetened beverages, posing a higher health risk than other UPF [178,179]. It is important to note that the composition of UPF subgroups can vary by context when the Nova UPF score metric is adapted [180], posing challenges for cross-country comparisons and complicating global monitoring efforts. The lack of comprehensive metrics to assess moderation more holistically highlights the need for the development of more metrics that can provide a fuller picture of children and adolescents’ dietary risks and help guide more effective policies and interventions.

An additional gap for these 5 identified metrics is the limited validation evidence pertinent to global monitoring, specifically in terms of test-retest reliability and sensitivity to change. These aspects are crucial for ensuring that metrics can reliably measure healthy diet subconstructs over time and accurately reflect changes in these subconstructs [13], whether due to interventions or natural variations in eating patterns across seasons, time, and contexts. Test-retest reliability means a metric will consistently produce the same results under similar conditions, reinforcing the confidence in their use for global monitoring. Sensitivity to change is equally important, as it determines a metric’s ability to detect meaningful changes in dietary intake, which is critical for evaluating the impact of nutrition actions. The absence of this robust evidence for the 4 child- and adolescent-focused metrics identified in this review raises concerns about their overall measurement accuracy and reliability. The reliability and sensitivity of the MDD-W, the basis for MDD-10, have been more rigorously evaluated among women of reproductive age, with several studies showing MDD-W to accurately reflect when changes in diet diversity did or did not occur in populations, in alignment with changes in nutrient intakes, across seasons or in response to interventions [[181], [182], [183], [184]]. The lack of similar evidence for child-focused diet metrics underscores a critical need for further research to validate these methods and metrics, particularly in terms of their consistency and responsiveness to dietary changes. Without this validation, the full utility of these metrics for monitoring the healthiness of diets among this age group remains uncertain.

Furthermore, the studies of the 5 metrics only investigated relationships among individuals within populations, whereas the primary interests for global monitoring are differences among, and change over time in, populations and subpopulations. Explicit evidence is needed to determine whether metrics feasible for global monitoring can adequately differentiate healthy diets of children and adolescents across countries and how child and adolescent diets within countries change over time.

Of the 5 metrics identified as suitable for global monitoring, 2 stem from prior existing metrics for adult populations: MDD-10 and the Adapted Nova UPF score. The Global Diet Quality Score is also a healthy diet metric for adults currently being adapted and validated for use among children and younger adolescents [[185], [186], [187]], which may be another promising option for global monitoring. Studies on the construct validity of both MDD-10 and the Adapted Nova UPF score suggest that metrics designed for assessing adult diets can be effectively adapted for use among children and adolescents, and this adaptation offers potential logistical and feasibility benefits for global monitoring efforts. For both MDD-10 and the Adapted Nova UPF score, the same food groups are included in the metrics for both adults and younger populations, facilitating measurement across different age groups within single platform of data collection. Furthermore, the questionnaire used for collecting MDD-10 data is the same as that used for MDD-W and is already integrated into widely implemented national population-based survey platforms, such as the Demographic and Health Surveys and Gallup World Poll (including older adolescents aged 15–19 y), as well as various national nutrition and health surveillance systems. These platforms would need to include younger age groups in their sampling, and potentially increase their sample sizes, to generate measurements of dietary diversity for children and adolescents. Although MDD-10 shows promise as an effective adaptation for assessing dietary diversity among children and adolescents, future research should focus on adapting metrics that capture other subconstructs of a healthy diet, such as moderation, to better support comprehensive monitoring.

Although this review primarily focused on identifying metrics for assessing healthy diets among children and adolescents, the methods used to measure these metrics are equally critical when considering their suitability for global monitoring, as the choice of method can significantly impact the accuracy and reliability of the data collected. One of the key methodological questions for dietary assessment among children and adolescents is the accuracy and reliability of child-reported compared with guardian-reported consumption information. Determining the appropriate age at which children and adolescents can reliably report their own dietary intake compared with when a parent or guardian should be the respondent is crucial [188]. This present review highlighted variations in age cutoffs among studies; studies among younger children and adolescents typically relied on parental reporting, the age at which children and adolescents became the primary respondent varied from as young as 7 y in Brazil [33] to as old as 16 y in Mexico [120]. Parental recall may also be subject to bias and inaccuracies, particularly as parents may not always be present during all meals or eating occasions outside the home [189] or may have difficulty estimating portion sizes consumed by children and adolescents.

Recent advancements in dietary assessment methods may offer solutions to enhance the accuracy of dietary data collection in children and adolescents. Technology-based methods, including smartphone applications, provide innovative ways to assess dietary intake using visual aids and web-based platforms that can ease dietary data collection [190,191]. Several of these methods have been shown to improve portion size estimation and reduce participant burden [192,193], making them potentially valuable for large-scale monitoring efforts among children and adolescents. More recently, the integration of artificial intelligence and machine learning into dietary assessment tools has opened up new possibilities for collecting dietary data with greater precision and regularity. These developments hold promise for increasing the feasibility of collecting more granular, quantitative dietary data at scale, an endeavor historically constrained by resource intensity and respondent fatigue. Although such technologies are advancing rapidly, their validation in diverse child and adolescent populations is still needed to confirm appropriateness for global use. The effectiveness of these technological methods, alongside traditional methods like 24-h recalls and FFQ, must be carefully considered when selecting metrics and methods for assessing children and adolescents’ diets on a global scale.

Among the strengths of this review is the systematic method employed in the literature search, which ensured a comprehensive identification of existing metrics and methods for assessing healthy diets among children and adolescents. Additionally, evidence captured through this search was augmented by interviews with key experts in the field, which not only facilitated the sourcing of additional grey literature but also provided valuable insights into the logistical feasibility of data collection for the 4 identified metrics. These expert interviews added a practical dimension to the review and aided the assessment of real-world feasibility of the identified metrics, which is often not presented in published literature. Nonetheless, this review has several limitations related to its methodology. One significant limitation is the exclusion of non–English literature, which may have resulted in the omission of relevant studies published in other languages, potentially biasing the findings toward English-speaking regions and authors. In addition, although the review followed a systematic and structured method to searching and evaluating the literature, the review was not registered in a protocol repository (e.g., PROSPERO), and duplicate screening, data extraction, and quality assessment were not possible for all papers. Despite these limitations, this review provides a valuable synthesis of current knowledge and identifies key gaps for future research.

Regardless of the increasing number of metrics available to assess the healthiness of diets among children and adolescents, few demonstrate the feasibility and adaptability required for global monitoring. Furthermore, those metrics that offer a lightweight, feasible, universal indicator primarily focus on dietary diversity, and metrics development and validation for other critical subconstructs of a healthy diet have lagged, such as the moderation of foods and nutrients associated with increased risk of noncommunicable diseases, a growing concern worldwide. There are also critical gaps in understanding the reliability and sensitivity of these metrics, and explicit investigation of validity to differentiate populations and changes in populations, which are essential for ensuring that these indicators can reliably track the healthiness of diets over time, accurately reflect the impact of interventions, and compare populations to one another.

It is imperative for research to address these gaps, with a focus on developing or adapting additional metrics to encompass a broader range of subconstructs of a healthy diet. Additionally, the exploration and validation of innovative, technology-based dietary assessment methods tailored for children and adolescents should be prioritized to enhance data accuracy and feasibility in global monitoring efforts. By strengthening the evidence base and expanding the scope of these methods, we can better support global initiatives aimed at providing guidance on monitoring to countries, improving child and adolescent diets, promoting healthier eating habits in the future, and enabling long-term well-being worldwide.

## Author contributions

The authors’ responsibilities were as follows – AMP, EAF, JCC: designed the research with input from all coauthors; AMP, HC: conducted research; AMP: analyzed data; AMP, HC: wrote the paper; AMP: had primary responsibility for final content; and all authors: read and approved the final manuscript.

## Data availability

Data described in the manuscript will be made available on reasonable request to the corresponding author.

## Funding

This work was supported, in whole or in part, by the Rockefeller Foundation (grant: 2022 FOD 024) and the Bill & Melinda Gates Foundation (grant: INV-063321). Under the grant conditions of the Bill & Melinda Foundation, a Creative Commons Attribution 4.0 Generic License has already been assigned to the Author Accepted Manuscript version that might arise from this submission.

## Declaration of generative AI and AI-assisted technologies in the writing process

During the preparation of this work, the author(s) used ChatGPT in order to provide a final copy-edit for grammar in sections of the manuscript. After using this tool/service, the author(s) reviewed and edited the content as needed and take(s) full responsibility for the content of the publication.

## Conflict of interest

The authors report no conflicts of interest.

## References

[1] Norris S.A., Frongillo E.A., Black M.M., Dong Y., Fall C., Lampl M. (2022). Nutrition in adolescent growth and development. Lancet.

[2] Black R.E., Victora C.G., Walker S.P., Bhutta Z.A., Christian P., De Onis M. (2013). Maternal and child undernutrition and overweight in low-income and middle-income countries. Lancet.

[3] Fanzo J., Miachon L. (2023). Harnessing the connectivity of climate change, food systems and diets: taking action to improve human and planetary health. Anthropocene.

[4] Popkin B.M., Corvalan C., Grummer-Strawn L.M. (2020). Dynamics of the double burden of malnutrition and the changing nutrition reality. Lancet..

[5] Phelps N.H., Singleton R.K., Zhou B., Heap R.A., Mishra A., Bennett J.E. (2024). Worldwide trends in underweight and obesity from 1990 to 2022: a pooled analysis of 3663 population-representative studies with 222 million children, adolescents, and adults. Lancet.

[6] Micha R., Coates J., Leclercq C., Charrondiere U.R., Mozaffarian D. (2018). Global dietary surveillance: data gaps and challenges. Food Nutr. Bull..

[7] Marshall S., Burrows T., Collins C.E. (2014). Systematic review of diet quality indices and their associations with health-related outcomes in children and adolescents. J. Hum. Nutr. Diet..

[8] Dalwood P., Marshall S., Burrows T.L., McIntosh A., Collins C.E. (2020). Diet quality indices and their associations with health-related outcomes in children and adolescents: an updated systematic review. Nutr. J..

[9] (2024). World Health Organization, Food and Agriculture Organization of the United Nations and United Nations Children’s Fund.

[10] Livingstone M.B.E., Robson P.J., Wallace J.M.W. (2004). Issues in dietary intake assessment of children and adolescents. Br. J. Nutr..

[11] Harmonizing and mainstreaming the measurement of healthy diets: technical expert meeting, Bellagio, Italy, 28 November-2 December 2022. Geneva: World Health Organization; 2023. Licence: CC BY-NC-SA 3.0 IGO.

[12] Verger E.O., Savy M., Martin-Prével Y., Coates J., Frongillo E., Neufeld L. (2023). Healthy diet metrics: a suitability assessment of indicators for global and national monitoring purposes.

[13] Kirkpatrick S.I., Baranowski T., Subar A.F., Tooze J.A., Frongillo E.A. (2019). Best practices for conducting and interpreting studies to validate self-report dietary assessment methods. J. Acad. Nutr. Diet..

[14] Miller V., Webb P., Micha R., Mozaffarian D. (2020). Defining diet quality: a synthesis of dietary quality metrics and their validity for the double burden of malnutrition. Lancet Planet. Health.

[15] Academy of Nutrition and Dietetics (2016). http://www.eatrightpro.org.

[16] Previdelli A.N., Andrade S.C., Pires M.M., Ferreira S.R., Fisberg R.M., Marchioni D.M. (2011). A revised version of the Healthy Eating Index for the Brazilian population. Rev. Saude Publica..

[17] Chamoli R., Jain M., Tyagi G. (2019). Reliability and validity of the Diet Quality Index for 7-9-year-old Indian children. Pediatr. Gastroenterol. Hepatol. Nutr..

[18] Nunez-Rivas H.P., Holst-Schumacher I., Campos-Saborio N. (2020). New diet quality index for children and adolescents in Costa Rica, Nutr. Hosp.

[19] Crilley E., Brownlee I., Defeyter M.A. (2021). The diet of children attending a holiday programme in the UK: adherence to UK food-based dietary guidelines and school food standards. Int. J. Environ. Res. Public Health..

[20] Gomez G., Fisberg R.M., Nogueira Previdelli A., Hermes Sales C., Kovalskys I., Fisberg M. (2019). Diet quality and diet diversity in eight Latin American countries: results from the Latin American Study of Nutrition and Health (ELANS). Nutrients.

[21] Micha R., Shulkin M.L., Peñalvo J.L., Khatibzadeh S., Singh G.M., Rao M. (2017). Etiologic effects and optimal intakes of foods and nutrients for risk of cardiovascular diseases and diabetes: systematic reviews and meta-analyses from the nutrition and chronic diseases expert group (NutriCoDE). PLOS ONE.

[22] Wu H., Yuan Y.Q., Wang Y.C., Zhou X.F., Liu S.J., Cai M.Q. (2021). The development of a Chinese Healthy Eating Index for school-age children and its application in children from China Health and Nutrition Survey. Int. J. Food Sci. Nutr..

[23] Aguilar Aldrete M.E., López-Toledo S., Caballero Avendano A., Villa Ruano N., Navarro Hernandez R.E., Flores Alvarado L.J. (2021). Association between nutritional risk markers and polymorphisms rs2291166 in TJP1 and VNTR (CAG)n in ATXN2 in an obese adolescent Mexican population, Endocrinol. Diabetes Nutr. (Engl. Ed.)..

[24] Temple J.L., Mansouri T., Andrade A.L.P., Ziegler A.M. (2023). The influence of relative reinforcing value of food, sensitization, energy intake and diet quality on zBMI change over two years in adolescents: a longitudinal cohort study. Nutrients.

[25] Gaona-Pineda E.B., Lopez-Olmedo N., Moreno-Macias H., Shamah-Levy T. (2024). Three approaches to assessing dietary quality in Mexican adolescents from 2006 to 2018 with data from national health and nutrition surveys. Public Health Nutr.

[26] Rodriguez-Mireles S., Lopez-Valcarcel B.G., Galdos-Arias P., Perez-Diaz E., Serra-Majem L. (2024). Socioeconomic disparities in diet and physical activity in children: evidence from well-child visit electronic health records in the Canary Islands, Spain. J. Epidemiol. Community Health..

[27] van der Velde L.A., Nguyen A.N., Schoufour J.D., Geelen A., Jaddoe V.W.V., Franco O.H. (2019). Diet quality in childhood: the Generation R Study. Eur. J. Nutr..

[28] Bjerregaard A.A., Halldorsson T.I., Tetens I., Olsen S.F. (2019). Mother’s dietary quality during pregnancy and offspring’s dietary quality in adolescence: follow-up from a national birth cohort study of 19,582 mother-offspring pairs. PLOS Med.

[29] Brownlee I.A., Low J., Duriraju N., Chun M., Ong J.X.Y., Tay M.E. (2019). Evaluation of the proximity of Singaporean children’s dietary habits to food-based dietary guidelines. Nutrients.

[30] Moraeus L., Lindroos A.K., Warensjö Lemming E., Mattisson I. (2020). Diet diversity score and healthy eating index in relation to diet quality and socio-demographic factors: results from a cross-sectional national dietary survey of Swedish adolescents. Public Health Nutr.

[31] Benetou V., Kanellopoulou A., Kanavou E., Fotiou A., Stavrou M., Richardson C. (2020). Diet-related behaviors and diet quality among school-aged adolescents living in Greece. Nutrients.

[32] Chacón V., Liu Q., Park Y., Rohloff P., Barnoya J. (2021). Diet quality, school attendance, and body weight status in adolescent girls in rural Guatemala. Ann. N. Y. Acad. Sci..

[33] Giacomelli S.C., de Assis M.A.A., de Andrade D.F., Schmitt J., Hinnig P.F., Borgatto A.F. (2021). Development of a food-based diet quality scale for Brazilian schoolchildren using item response theory. Nutrients.

[34] Jailani M., Elias S.M., Rajikan R. (2021). The new standardized Malaysian Healthy Eating Index. Nutrients.

[35] Koivuniemi E., Nuutinen O., Riskumaki M., Vahlberg T., Laitinen K. (2021). Development of a stand-alone index for the assessment of diet quality in elementary school-aged children. Public Health Nutr.

[36] Wang X., Xu Y., Tan B., Duan R., Shan S., Zeng L. (2022). Development of the Chinese preschooler dietary index: a tool to assess overall diet quality. BMC Public Health.

[37] van Meijeren-van Lunteren A.W., Voortman T., Wolvius E.B., Kragt L. (2023). Adherence to dietary guidelines and dental caries among children: a longitudinal cohort study. Eur. J. Public Health..

[38] Alexy U., Kersting M., Schultze-Pawlitschko V. (2003). Two approaches to derive a proposal for added sugars intake for German children and adolescents. Public Health Nutr.

[39] Alexy U., Sichert-Hellert W., Kersting M., Lausen B., Schöch G. (1999). Development of scores to measure the effects of nutrition counselling on the overall diet: a pilot study in children and adolescents. Eur. J. Nutr..

[40] Absolon J.S., Wearring G.A., Behme M.T. (1988). Dietary quality and eating patterns of adolescent girls in Southwestern Ontario. J. Nutr. Educ..

[41] Lazarou C., Panagiotakos D.B., Matalas A.L. (2009). Foods E-KINDEX: a dietary index associated with reduced blood pressure levels among young children: the CYKIDS Study. J. Am. Diet. Assoc..

[42] Cheng G., Duan R., Kranz S., Libuda L., Zhang L. (2016). Development of a dietary index to assess overall diet quality for Chinese school-aged children: the Chinese Children Dietary Index. J. Acad. Nutr. Diet..

[43] Arvidsson L., Eiben G., Hunsberger M., De Bourdeaudhuij I., Molnar D., Jilani H. (2017). Bidirectional associations between psychosocial well-being and adherence to healthy dietary guidelines in European children: prospective findings from the IDEFICS study. BMC Public Health.

[44] Röytiö H., Jaakkola J., Hoppu U., Poussa T., Laitinen K. (2015). Development and evaluation of a stand-alone index for the assessment of small children’s diet quality. Public Health Nutr.

[45] Kleiser C., Mensink G.B., Scheidt-Nave C., Kurth B.M. (2009). HuSKY: a healthy nutrition score based on food intake of children and adolescents in Germany. Br. J. Nutr..

[46] Kohlboeck G., Sausenthaler S., Standl M., Koletzko S., Bauer C.P., von Berg A. (2012). Food intake, diet quality and behavioral problems in children: results from the GINI-plus/LISA-plus studies. Ann. Nutr. Metab..

[47] Manios Y., Kourlaba G., Grammatikaki E., Androutsos O., Moschonis G., Roma-Giannikou E. (2010). Development of a diet–lifestyle quality index for young children and its relation to obesity: the Preschoolers Diet–Lifestyle Index. Public Health Nutr.

[48] Manios Y., Moschonis G., Papandreou C., Politidou E., Naoumi A., Peppas D. (2015). Revised Healthy Lifestyle-Diet Index and associations with obesity and iron deficiency in schoolchildren: the Healthy Growth Study. J. Hum. Nutr. Diet..

[49] Magriplis E., Farajian P., Risvas G., Panagiotakos D., Zampelas A. (2015). Newly derived children-based food index. An index that may detect childhood overweight and obesity. Int. J. Food Sci. Nutr..

[50] Yannakoulia M., Karayiannis D., Terzidou M., Kokkevi A., Sidossis L.S. (2004). Nutrition-related habits of Greek adolescents. Eur. J. Clin. Nutr..

[51] Manios Y., Kourlaba G., Grammatikaki E., Koubitski A., Siatitsa P.E., Vandorou A. (2010). Development of a lifestyle–diet quality index for primary schoolchildren and its relation to insulin resistance: the Healthy Lifestyle–Diet Index. Eur. J. Clin. Nutr..

[52] Chiplonkar S.A., Tupe R. (2010). Development of a diet quality index with special reference to micronutrient adequacy for adolescent girls consuming a lacto-vegetarian diet. J. Am. Diet. Assoc..

[53] Perry C.P., Keane E., Layte R., Fitzgerald A.P., Perry I.J., Harrington J.M. (2015). The use of a dietary quality score as a predictor of childhood overweight and obesity. BMC Public Health.

[54] Choi Y., You Y., Go K.A., Tserendejid Z., You H.J., Lee J.E. (2013). The prevalence of obesity and the level of adherence to the Korean Dietary Action Guides in Korean preschool children. Nutr. Res. Pract..

[55] Rezali F.W., Chin Y.S., Mohd Shariff Z., Mohd Yusof B.N., Sanker K., Woon F.C. (2015). Evaluation of diet quality and its associated factors among adolescents in Kuala Lumpur, Malaysia. Nutr. Res. Pract..

[56] Wong J.E., Parnell W.R., Howe A.S., Black K.E., Skidmore P.M. (2013). Development and validation of a food-based diet quality index for New Zealand adolescents. BMC Public Health.

[57] Handeland K., Kjellevold M., Wik Markhus M., Eide Graff I., Frøyland L., Lie Ø. (2016). A diet score assessing Norwegian adolescents’ adherence to dietary recommendations—development and test-retest reproducibility of the score. Nutrients.

[58] Kennedy G.L., Pedro M.R., Seghieri C., Nantel G., Brouwer I. (2007). Dietary diversity score is a useful indicator of micronutrient intake in non-breast-feeding Filipino children. J. Nutr..

[59] Vilela S., Oliveira A., Ramos E., Moreira P., Barros H., Lopes C. (2014). Association between energy-dense food consumption at 2 years of age and diet quality at 4 years of age. Br. J. Nutr..

[60] da Costa M.P., Durao C., Lopes C., Vilela S. (2019). Adherence to a healthy eating index from pre-school to school age and its associations with sociodemographic and early life factors. Br. J. Nutr..

[61] Maia I., Oliveira A., Severo M., Santos A.C. (2023). Associations between children’s reports of food insecurity and dietary patterns: findings from the generation XXI birth cohort. Br. J. Nutr..

[62] Vilela S., Muresan I., Correia D., Severo M., Lopes C. (2020). The role of socio-economic factors in food consumption of Portuguese children and adolescents: results from the National Food, Nutrition and Physical Activity Survey 2015–2016. Br. J. Nutr..

[63] Crombie I.K., Kiezebrink K., Irvine L., Wrieden W.L., Swanson V., Power K. (2009). What maternal factors influence the diet of 2-year-old children living in deprived areas? A cross-sectional survey. Public Health Nutr.

[64] Serra-Majem L., Ribas L., Ngo J., Ortega R.M., García A., Pérez-Rodrigo C. (2004). Food, youth and the Mediterranean diet in Spain. Development of KIDMED, Mediterranean Diet Quality Index in children and adolescents. Public Health Nutr.

[65] Martin-Calvo N., Chavarro J.E., Falbe J., Hu F.B., Field A.E. (2016). Adherence to the Mediterranean dietary pattern and BMI change among US adolescents. Int. J. Obes. (Lond.)..

[66] Altavilla C., Caballero-Perez P. (2019). An update of the KIDMED questionnaire, a Mediterranean Diet Quality Index in children and adolescents. Public Health Nutr.

[67] Simon M.I.S.D.S., Forte G.C., Marostica P.J.C. (2020). Translation and cultural adaptation of the mediterranean diet quality index in children and adolescents. Rev. Paul. Pediatr..

[68] Mariscal-Arcas M., Velasco J., Monteagudo C., Caballero-Plasencia M.A., Lorenzo-Tovar M.L., Olea-Serrano F. (2010). Comparison of methods to evaluate the quality of the Mediterranean diet in a large representative sample of young people in Southern Spain. Nutr. Hosp..

[69] Monge-Rojas R., O’Neill J., Lee-Bravatti M., Mattei J. (2021). A traditional Costa Rican adolescents’ diet score is a valid tool to capture diet quality and identify sociodemographic groups with suboptimal diet. Front. Public Health.

[70] Mariscal-Arcas M., Romaguera D., Rivas A., Feriche B., Pons A., Tur J.A. (2007). Diet quality of young people in southern Spain evaluated by a Mediterranean adaptation of the Diet Quality Index-International (DQI-I). Br. J. Nutr..

[71] Chen Y.C., Huang Y.C., Lo Y.C., Wu H.J., Wahlqvist M.L., Lee M.S. (2018). Secular trend towards ultra-processed food consumption and expenditure compromises dietary quality among Taiwanese adolescents. Food Nutr. Res..

[72] Jessri M., Ng A.P., L’Abbé M.R. (2017). Adapting the Healthy Eating Index 2010 for the Canadian population: evidence from the Canadian Community Health Survey. Nutrients.

[73] Kennedy E.T., Ohls J., Carlson S., Fleming K. (1995). The Healthy Eating Index: design and applications. J. Am. Diet. Assoc..

[74] Glanville N.T., McIntyre L. (2006). Diet quality of Atlantic families headed by single mothers. Can. J. Diet. Pract. Res..

[75] Rauber F., da Costa Louzada M.L., Vitolo M.R. (2014). Healthy Eating Index measures diet quality of Brazilian children of low socioeconomic status. J. Am. Coll. Nutr..

[76] Feskanich D., Rockett H.R., Colditz G.A. (2004). Modifying the healthy eating index to assess diet quality in children and adolescents. J. Am. Diet. Assoc..

[77] Chiang P.H., Wahlqvist M.L., Lee M.S., Huang L.Y., Chen H.H., Huang S.T. (2011). Fast-food outlets and walkability in school neighbourhoods predict fatness in boys and height in girls: a Taiwanese population study. Public Health Nutr.

[78] Lee M.S., Huang L.Y., Chang Y.H., Huang S.T., Yu H.L., Wahlqvist M.L. (2012). Lower birth weight and diet in Taiwanese girls more than boys predicts learning impediments. Res. Dev. Disabil..

[79] Protudjer J.L., Sevenhuysen G.P., Ramsey C.D., Kozyrskyj A.L., Becker A.B. (2012). Low vegetable intake is associated with allergic asthma and moderate-to-severe airway hyperresponsiveness. Pediatr. Pulmonol..

[80] Hooshmand F., Asghari G., Yuzbashian E., Mahdavi M., Mirmiran P., Azizi F. (2018). Modified healthy eating index and incidence of metabolic syndrome in children and adolescents: Tehran lipid and glucose study. J. Pediatr..

[81] Switkowski K.M., Aris I.M., Gingras V., Oken E., Young J.G. (2022). Estimated causal effects of complementary feeding behaviors on early childhood diet quality in a US cohort. Am. J. Clin. Nutr..

[82] Guenther P.M., Reedy J., Krebs-Smith S.M. (2008). Development of the Healthy Eating Index-2005. J. Am. Diet. Assoc..

[83] Woodruff S.J., Hanning R.M. (2010). Development and implications of a revised Canadian Healthy Eating Index (HEIC-2009). Public Health Nutr.

[84] Guenther P.M., Casavale K.O., Reedy J., Kirkpatrick S.I., Hiza H.A., Kuczynski K.J. (2013). Update of the Healthy Eating Index: HEI-2010. J. Acad. Nutr. Diet..

[85] Horta P.M., Verly Junior E., Santos L.C.D. (2019). Usual diet quality among 8- to 12-year-old Brazilian children. Cad. Saude Publica..

[86] Caferoglu Z., Erdal B., Hatipoglu N., Kurtoglu S. (2022). The effects of diet quality and dietary acid load on insulin resistance in overweight children and adolescents. Endocrinol. Diabetes Nutr. (Engl. Ed.)..

[87] Kranz S., Siega-Riz A.M., Herring A.H. (2004). Changes in diet quality of American preschoolers between 1977 and 1998. Am. J. Public Health..

[88] Kranz S., Hartman T., Siega-Riz A.M., Herring A.H. (2006). A diet quality index for American preschoolers based on current dietary intake recommendations and an indicator of energy balance. J. Am. Diet. Assoc..

[89] Keshani P., Salehi M., Kaveh M.H., Faghih S. (2018). Self-efficacy and cues to action: two main predictors of modified version of diet quality index in Iranian adolescents. Progr. Nutr..

[90] Cox D.R., Skinner J.D., Carruth B.R., Moran J., Houck K.S. (1997). A Food Variety Index for Toddlers (VIT): development and application. J. Am. Diet. Assoc..

[91] Skinner J.D., Carruth B.R., Houck K.S., Bounds W., Morris M., Cox D.R. (1999). Longitudinal study of nutrient and food intakes of white preschool children aged 24 to 60 months. J. Am. Diet. Assoc..

[92] Jones L., Moschonis G., Oliveira A., de Lauzon-Guillain B., Manios Y., Xepapadaki P. (2015). The influence of early feeding practices on healthy diet variety score among pre-school children in four European birth cohorts. Public Health Nutr.

[93] Barros L., Lopes C., Oliveira A. (2019). Child and family characteristics are associated with a dietary variety index in 4-year-old children from the Generation XXI cohort. Nutr. Res..

[94] Drewnowski A. (2009). Defining nutrient density: development and validation of the Nutrient Rich Foods Index. J. Am. Coll. Nutr..

[95] Falciglia G.A., Troyer A.G., Couch S.C. (2004). Dietary variety increases as a function of time and influences diet quality in children. J. Nutr. Educ. Behav..

[96] Günther A.L., Liese A.D., Bell R.A., Dabelea D., Lawrence J.M., Rodriguez B.L. (2009). Association between the dietary approaches to hypertension diet and hypertension in youth with diabetes mellitus. Hypertension.

[97] Kyttälä P., Erkkola M., Lehtinen-Jacks S., Ovaskainen M.L., Uusitalo L., Veijola R. (2014). Finnish Children Healthy Eating Index (FCHEI) and its associations with family and child characteristics in pre-school children. Public Health Nutr.

[98] Gedrich K., Karg G. (2001).

[99] Golley R.K., Hendrie G.A., McNaughton S.A. (2011). Scores on the dietary guideline index for children and adolescents are associated with nutrient intake and socio-economic position but not adiposity. J. Nutr..

[100] Lioret S., McNaughton S.A., Cameron A.J., Crawford D., Campbell K.J., Cleland V.J. (2014). Three-year change in diet quality and associated changes in BMI among schoolchildren living in socio-economically disadvantaged neighbourhoods. Br. J. Nutr..

[101] Jacka F.N., Kremer P.J., Leslie E.R., Berk M., Patton G.C., Toumbourou J.W. (2010). Associations between diet quality and depressed mood in adolescents: results from the Australian Healthy Neighbourhoods Study. Aust. N. Z. J. Psychiatry.

[102] Li J., O’Sullivan T., Johnson S., Stanley F., Oddy W. (2012). Maternal work hours in early to middle childhood link to later adolescent diet quality. Public Health Nutr.

[103] Scott J.A., Chih T.Y., Oddy W.H. (2012). Food variety at 2 years of age is related to duration of breastfeeding. Nutrients.

[104] Kunaratnam K., Halaki M., Wen L.M., Baur L.A., Flood V.M. (2018). Reliability and comparative validity of a Diet Quality Index for assessing dietary patterns of preschool-aged children in Sydney, Australia. Eur. J. Clin. Nutr..

[105] Huybrechts I., Vereecken C., De Bacquer D., Vandevijvere S., Van Oyen H., Maes L. (2010). Reproducibility and validity of a diet quality index for children assessed using a FFQ. Br. J. Nutr..

[106] Vyncke K.E., Huybrechts I., Dallongeville J., Mouratidou T., Van Winckel M.A., Cuenca-García M. (2013). Intake and serum profile of fatty acids are weakly correlated with global dietary quality in European adolescents. Nutrition.

[107] De Vriendt T., Clays E., Huybrechts I., De Bourdeaudhuij I., Moreno L.A., Patterson E. (2012). European adolescents’ level of perceived stress is inversely related to their diet quality: the Healthy Lifestyle in Europe by Nutrition in Adolescence study. Br. J. Nutr..

[108] Sabbe D., De Bourdeaudhuij I., Legiest E., Maes L. (2008). A cluster-analytical approach towards physical activity and eating habits among 10-year-old children. Health Educ. Res..

[109] Molina Mdel C., Lopéz P.M., Faria C.P., Cade N.V., Zandonade E. (2010). Socioeconomic predictors of child diet quality. Rev. Saude Publica..

[110] Yuan Y.Q., Li F., Dong R.H., Chen J.S., He G.S., Li S.G. (2017). The development of a Chinese healthy eating index and its application in the general population. Nutrients.

[111] Burrows T.L., Collins K., Watson J., Guest M., Boggess M.M., Neve M. (2014). Validity of the Australian Recommended Food Score as a diet quality index for pre-schoolers. Nutr. J..

[112] Gasser C.E., Kerr J.A., Mensah F.K., Wake M. (2017). Stability and change in dietary scores and patterns across six waves of the Longitudinal Study of Australian Children. Br. J. Nutr..

[113] Chamoli R., Vandana, Jain M. (2019). Reliability estimation and pilot testing of diet quality assessment tool for Indian children. Indian J. Public Health Res. Dev..

[114] Bell L., Manson A., Zarnowiecki D., Tan S.N., Byrne R., Taylor R. (2024). Development and validation of a short dietary questionnaire for assessing obesity-related dietary behaviours in young children, Matern. Child Nutr..

[115] Delshad M., Beck K.L., von Hurst P.R., Mugridge O., Conlon C.A. (2019). The validity and reliability of the Dietary Index for a Child’s Eating in 2–8-year old children living in New Zealand, Matern. Child Nutr..

[116] Jarman M., Vashi N., Angus A., Bell R.C., Giesbrecht G.F., APrON study team (2020). Development of a diet quality index to assess adherence to Canadian dietary recommendations in 3-year-old children. Public Health Nutr.

[117] Lyu J.L., Liu Z., Zhou S., Feng X.X., Lin Y., Gao A.Y. (2022). The effect of a multifaceted intervention on dietary quality in schoolchildren and the mediating effect of dietary quality between intervention and changes in adiposity indicators: a cluster randomized controlled trial. Nutrients.

[118] Switkowski K.M., Kronsteiner-Gicevic S., Rifas-Shiman S.L., Lightdale J.R., Oken E. (2024). Evaluation of the Prime Diet Quality Score from early childhood through mid-adolescence. J. Nutr..

[119] Diop L., Becquey E., Turowska Z., Huybregts L., Ruel M.T., Gelli A. (2021). Standard minimum dietary diversity indicators for women or infants and young children are good predictors of adequate micronutrient intakes in 24-59-month-old children and their nonpregnant nonbreastfeeding mothers in rural Burkina Faso. J. Nutr..

[120] Rodriguez-Ramirez S., Sanchez-Pimienta T.G., Batis C., Cediel G., Marron-Ponce J.A. (2021). Minimum dietary diversity in Mexico: establishment of cutoff point to predict micronutrients adequacy. Eur. J. Clin. Nutr..

[121] Monge-Rojas R., Vargas-Quesada R., Marron-Ponce J.A., Sanchez-Pimienta T.G., Batis C., Rodriguez-Ramirez S. (2024). Exploring differences in dietary diversity and micronutrient adequacy between Costa Rican and Mexican adolescents. Children (Basel).

[122] Hanley-Cook G.T., Hoogerwerf S., Parraguez J.P., Gie S.M., Holmes B.A. (2024). Minimum dietary diversity for adolescents: multicountry analysis to define food group thresholds predicting micronutrient adequacy among girls and boys aged 10-19 years, Curr. Dev. Nutr..

[123] Arimond M., Wiesmann D., Rodríguez Ramírez S., Shamah Levy T., Ma S., Zou Z. (2021 June). https://www.gainhealth.org/sites/default/files/publications/documents/gain-discussion-paper-series-9-food-group-diversity-and-nutrient-adequacy.pdf.

[124] Madzorera I., Bromage S., Mwanyika-Sando M., Vandormael A., Sherfi H., Worku A. (2025). Dietary intake and quality for young adolescents in sub-Saharan Africa: status and influencing factors, Matern. Child Nutr..

[125] Matthews L.A., Rovio S.P., Jaakkola J.M., Niinikoski H., Lagstrom H., Jula A. (2019). Longitudinal effect of 20-year infancy-onset dietary intervention on food consumption and nutrient intake: the randomized controlled STRIP study. Eur. J. Clin. Nutr..

[126] Hamner H.C., Moore L.V. (2020). Dietary quality among children from 6 months to 4 years, NHANES 2011-2016. Am. J. Clin. Nutr..

[127] Harris H.R., Willett W.C., Vaidya R.L., Michels K.B. (2016). Adolescent dietary patterns and premenopausal breast cancer incidence. Carcinogenesis.

[128] Tarazona-Meza C.E., Hanson C., Pollard S.L., Romero Rivero K.M., Galvez Davila R.M., Talegawkar S. (2020). Dietary patterns and asthma among Peruvian children and adolescents. BMC Pulm. Med..

[129] Mora-Garcia G., Ruiz-Diaz M.S., Villegas R., Garcia-Larsen V. (2020). Changes in diet quality over 10 years of nutrition transition in Colombia: analysis of the 2005 and 2015 nationally representative cross-sectional surveys. Int. J. Public Health..

[130] Askari M., Daneshzad E., Bellissimo N., Suitor K., Dorosty-Motlagh A.R., Azadbakht L. (2021). Food quality score and anthropometric status among 6-year-old children: a cross-sectional study. Int. J. Clin. Pract.

[131] Tarro S., Lahdenpera M., Vahtera J., Pentti J., Lagstrom H. (2022). Diet quality in preschool children and associations with individual eating behavior and neighborhood socioeconomic disadvantage. The STEPS Study. Appetite.

[132] Rolands M.R., Toh J.Y., Sugianto R., Yuan W.L., Lee Y.S., Tan K.H. (2023). Development and evaluation of a diet quality index for preschool-aged children in an Asian population: the growing up in Singapore towards healthy outcomes cohort. J. Acad. Nutr. Diet..

[133] Wu S., Xiu X., Qian Q. (2023). Associations between dietary patterns and physical activity with physical fitness among adolescents in Shandong Province, China: a cross-sectional study. Nutrients.

[134] Vinke P.C., Luitjens M.H.H.S., Blijleven K.A., Navis G., Kromhout D., Corpeleijn E. (2021). Nutrition beyond the first 1000 days: diet quality and 7-year change in BMI and overweight in 3-year old children from the Dutch GECKO Drenthe birth cohort. J. Dev. Orig. Health Dis..

[135] Shatenstein B., Abu-Shaaban D., Pascual M.L., Kark J.D. (1996). Dietary adequacy among urban and semi-rural schoolchildren in Gaza. Ecol. Food Nutr..

[136] Rohde J.F., Larsen S.C., Ängquist L., Olsen N.J., Stougaard M., Mortensen E.L. (2017). Effects of the healthy start randomized intervention on dietary intake among obesity-prone normal-weight children. Public Health Nutr.

[137] Henriksson P., Cuenca-García M., Labayen I., Esteban-Cornejo I., Henriksson H., Kersting M. (2017). Diet quality and attention capacity in European adolescents: the Healthy Lifestyle in Europe by Nutrition in Adolescence (HELENA) study. Br. J. Nutr..

[138] Haapala E.A., Eloranta A.M., Venäläinen T., Jalkanen H., Poikkeus A.M., Ahonen T. (2017). Diet quality and academic achievement: a prospective study among primary school children. Eur. J. Nutr..

[139] Mohseni-Takalloo S., Hosseini-Esfahani F., Mirmiran P., Azizi F. (2016). Associations of pre-defined dietary patterns with obesity associated phenotypes in Tehranian adolescents. Nutrients.

[140] Kuriyama N., Murakami K., Livingstone M.B.E., Okubo H., Kobayashi S., Suga H. (2016). Development of a food-based diet quality score for Japanese: associations of the score with nutrient intakes in young, middle-aged and older Japanese women. J. Nutr. Sci..

[141] Hu T., Jacobs D.R., Larson N.I., Cutler G.J., Laska M.N., Neumark-Sztainer D. (2016). Higher diet quality in adolescence and dietary improvements are related to less weight gain during the transition from adolescence to adulthood. J. Pediatr..

[142] Marshall S., Watson J., Burrows T., Guest M., Collins C.E. (2012). The development and evaluation of the Australian child and adolescent recommended food score: a cross-sectional study. Nutr. J..

[143] Correa-Madrid M.C., Correa Guzmán N., Bergeron G., Restrepo-Mesa S.L., Cediel G. (2023). Validation of the NOVA score for the consumption of ultra-processed foods by young women of Medellín, Colombia. Ann. N. Y. Acad. Sci..

[144] Chen Y.C., Lo Y.C., Wu H.Y., Huang Y.C. (2024). Adherence to dietary guidelines associated with lower medical service utilization in preschoolers: a longitudinal study, Nutr. Diabetes.

[145] Ronca D.B., Blume C.A., Cureau F.V., Camey S.A., Leotti V.B., Drehmer M. (2020). Diet quality index for Brazilian adolescents: the ERICA study. Eur. J. Nutr..

[146] Shinsugi C., Tani Y., Kurotani K., Takimoto H., Ochi M., Fujiwara T. (2020). Change in growth and diet quality among preschool children in Tokyo, Japan. Nutrients.

[147] World Health Organization (2008). Indicators for assessing infant and young child feeding practices: Part 1: definitions. World Health Organization.

[148] FAO (2021). Minimum Dietary Diversity for Women.

[149] Costa C.D.S., Faria F.R., Gabe K.T., Sattamini I.F., Khandpur N., Leite F.H.M. (2021). Nova score for the consumption of ultra-processed foods: description and performance evaluation in Brazil. Rev. Saude Publica..

[150] Wiafe M.A., Apprey C., Annan R.A. (2023). Dietary diversity and nutritional status of adolescents in rural Ghana. Nutr. Metab. Insights..

[151] de Castro-Mendes F., Cunha P., Paciencia I., Cavaleiro Rufo J., Farraia M., Silva D. (2021). The influence of eating at home on dietary diversity and airway inflammation in Portuguese school-aged children. Int. J. Environ. Res. Public Health..

[152] Getacher L., Ademe B.W., Belachew T. (2023). Double burden of malnutrition and its associated factors among adolescents in Debre Berhan Regiopolitan City, Ethiopia: a multinomial regression model analysis. Front. Nutr..

[153] Islam M.R., Rahman S.M., Selling K., Nasanen-Gilmore P., Kippler M., Kajantie E. (2023). Dietary patterns and indicators of cardiometabolic risk among rural adolescents: a cross-sectional study at 15-year follow-up of the MINIMAT cohort. Front. Nutr..

[154] Mank I., De Neve J.W., Mauti J., Gyengani G.A., Some P.A., Shinde S. (2022). Prevalence of obesity and anemia among early adolescents in junior secondary schools: a cross-sectional study in Ouagadougou, Burkina Faso. J. Sch. Health.

[155] Mersha J., Tariku A., Gonete K.A. (2021). Undernutrition and associated factors among school adolescent girls attending schools in Mirab-Armachiho District, Northwest Ethiopia. Ecol. Food Nutr..

[156] Partap U., Tadesse A.W., Shinde S., Sherfi H., Mank I., Mwanyika-Sando M. (2025). Burden and determinants of anaemia among in-school young adolescents in Ethiopia, Sudan and Tanzania, Matern. Child Nutr..

[157] Wemakor A., Kwaako M., Abdul-Rahman A. (2023). Nutritional, health and socio-demographic determinants of anaemia in adolescent girls in Kumbungu District, Ghana. BMC Nutr.

[158] Aboagye R.G., Kugbey N., Ahinkorah B.O., Seidu A.A., Cadri A., Bosoka S.A. (2022). Nutritional status of school children in the South Tongu District, Ghana. PLOS ONE.

[159] Motadi S.A., Zuma M.K., Freeland-Graves J.H., Gertrude Mbhenyane X. (2023). Dietary diversity and nutritional status of children attending early childhood development centres in Vhembe District, Limpopo province, South Africa. J. Nutr. Sci..

[160] Yazew T. (2022). Are dietary diversity and food insecurity associated with nutritional status of children in Western Oromia, Ethiopia?. J. Biol. Today’s World..

[161] Bliznashka L., Perumal N., Yousafzai A., Sudfeld C. (2022). Diet and development among children aged 36-59 months in low-income countries. Arch. Dis. Child..

[162] Mak T.N., Angeles-Agdeppa I., Lenighan Y.M., Capanzana M.V., Montoliu I. (2019). Diet diversity and micronutrient adequacy among Filipino school-age children. Nutrients.

[163] Torrico J.C. (2021). Dietary diversity score as an indicator of micronutrient intake in Filipino children and adolescents, Asia Pac. J. Clin. Nutr..

[164] Dogui D., Doggui R., El Ati J., El Ati-Hellal M. (2021). Association between overweight and diet diversity score: a cross-sectional study conducted among Tunisian children. Children (Basel).

[165] Demmler K.M., Beal T., Ghadirian M.Z., Neufeld L.M. (2023). Characteristics of global data on adolescent’s dietary intake: a systematic scoping review. Curr. Dev. Nutr..

[166] Kupka R., Siekmans K., Beal T. (2020). The diets of children: overview of available data for children and adolescents. Glob. Food Sec..

[167] Hanley-Cook G.T., Gie S.M., Parraguez J.P., Hoogerwerf S., Padula de Quadros V., Balcerzak A. (2024). Cross-context equivalence and agreement of healthy diet metrics for national and global monitoring: a multicountry analysis of cross-sectional quantitative 24-hour dietary intake studies. Am. J. Clin. Nutr..

[168] Diop L., Gelli A., Huybregts L., Arsenault J.E., Bliznashka L., Boy E. (2024). The minimum dietary diversity for women indicator can be extended to children and adolescents aged 4-15 years as a proxy population indicator for good micronutrient adequacy of diets in low- and middle-income countries. Curr. Dev. Nutr..

[169] Nguyen P.H., Huybregts L., Sanghvi T.G., Tran L.M., Frongillo E.A., Menon P. (2018). Dietary diversity predicts the adequacy of micronutrient intake in pregnant adolescent girls and women in Bangladesh, but use of the 5-group cutoff poorly identifies individuals with inadequate intake. J. Nutr..

[170] Trijsburg L., Talsma E.F., de Vries J.H.M., Kennedy G., Kuijsten A., Brouwer I.D. (2019). Diet quality indices for research in low- and middle-income countries: a systematic review. Nutr. Rev..

[171] Christian P., Smith E.R. (2018). Adolescent undernutrition: global burden, physiology, and nutritional risks, Ann. Nutr. Metab..

[172] World Health Organization, Food and Agriculture Organization of the United Nations (2024). What are healthy diets? WHO.

[173] Mendonça R.D., Pimenta A.M., Gea A., de la Fuente-Arrillaga C., Martinez-Gonzalez M.A., Lopes A.C. (2016). Ultraprocessed food consumption and risk of overweight and obesity: the University of Navarra Follow-Up (SUN) cohort study. Am. J. Clin. Nutr..

[174] de Oliveira P.G., de Sousa J.M., Assunção D.G.F., de Araujo E.K.S., Bezerra D.S., Dametto J.F.D.S. (2022). Impacts of consumption of ultra-processed foods on the maternal-child health: a systematic review. Front. Nutr..

[175] Srour B., Fezeu L.K., Kesse-Guyot E., Allès B., Méjean C., Andrianasolo R.M. (2019). Ultra-processed food intake and risk of cardiovascular disease: prospective cohort study (NutriNet-Santé). BMJ.

[176] García-Blanco L., de la O V., Santiago S., Pouso A., Martínez-González M.Á., Martín-Calvo N. (2023). High consumption of ultra-processed foods is associated with increased risk of micronutrient inadequacy in children: the SENDO project. Eur. J. Pediatr..

[177] Rousham E.K., Goudet S., Markey O., Griffiths P., Boxer B., Carroll C. (2022). Unhealthy food and beverage consumption in children and risk of overweight and obesity: a systematic review and meta-analysis. Adv. Nutr..

[178] Mendoza K., Smith-Warner S.A., Rossato S.L., Khandpur N., Manson J.E., Qi L. (2024). Ultra-processed foods and cardiovascular disease: analysis of three large US prospective cohorts and a systematic review and meta-analysis of prospective cohort studies. Lancet Reg. Health Am.

[179] Fang Z., Rossato S.L., Hang D., Khandpur N., Wang K., Lo C.H. (2024). Association of ultra-processed food consumption with all cause and cause specific mortality: population based cohort study. BMJ.

[180] Kébé S.D., Diouf A., Sylla P.M.D.D., Kane K., Dos Santos Costa C., Leite F.H.M. (2024). Assessment of ultra processed foods consumption in Senegal: validation of the Nova-UPF screener. Arch. Public Health.

[181] Nguyen P.H., Kachwaha S., Tran L.M., Avula R., Young M.F., Ghosh S. (2021). Strengthening nutrition interventions in antenatal care services affects dietary intake, micronutrient intake, gestational weight gain, and breastfeeding in Uttar Pradesh, India: results of a cluster-randomized program evaluation. J. Nutr..

[182] Ghosh-Jerath S., Kapoor R., Bandhu A., Singh A., Downs S., Fanzo J. (2022). Indigenous foods to address malnutrition: an inquiry into the diets and nutritional status of women in the indigenous community of Munda Tribes of Jharkhand, India. Curr. Dev. Nutr..

[183] Boedecker J., Odhiambo Odour F., Lachat C., Van Damme P., Kennedy G., Termote C. (2019). Participatory farm diversification and nutrition education increase dietary diversity in Western Kenya, Matern. Child Nutr..

[184] Koeryaman M.T., Pallikadavath S., Ryder I.H., Kandala N. (2023). The effectiveness of a web-based application for a balanced diet and healthy weight among Indonesian pregnant women: randomized controlled trial, JMIR Form. Res.

[185] Ali N.B., Arsenault J.E., Castellanos-Gutierrez A., Moursi M., Deitchler M., Batis C. (2025). Development and validation of the Global Diet Quality Score (GDQS) for children 24 to 59 months of age. Nutr. Rev..

[186] Batis C., Castellanos-Gutierrez A., Ali N.B., Arsenault J.E., Atayde A.M.P., Bromage S. (2025). Validation of the Global Diet Quality Score (GDQS) among children 10 to 14 years of age. Nutr. Rev..

[187] Arsenault J.E., Ali N.B., Atayde A.M.P., Batis C., Becquey E., Bromage S. (2025). Development and validation of the Global Diet Quality Score (GDQS) for children 5 to 9 years of age. Nutr. Rev..

[188] Callahan E., National Academies of Sciences (2022). Proceedings of a workshop series.

[189] Gewa C.A., Murphy S.P., Neumann C.G. (2007). Out-of-home food intake is often omitted from mothers’ recalls of school children’s intake in rural Kenya. J. Nutr..

[190] Baghlaf K. (2022). Traditional dietary assessment tools verses technology-based methods in children and adolescence. An updated review: traditional and technological diet assessment methods. Progr. Nutr..

[191] Mahal S., Kucha C., Kwofie E.M., Ngadi M. (2024). A systematic review of dietary data collection methodologies for diet diversity indicators. Front. Nutr..

[192] Bradley J., Simpson E., Poliakov I., Matthews J.N., Olivier P., Adamson A.J. (2016). Comparison of INTAKE24 (an Online 24-h dietary recall tool) with interviewer-led 24-h recall in 11–24 year-old. Nutrients.

[193] Foster E., Hawkins A., Simpson E., Adamson A.J. (2014). Developing an interactive portion size assessment system (IPSAS) for use with children. J. Hum. Nutr. Diet..

